# Unveiling Shared Genetic Architectures and Causality: Intestinal Diseases and Neurological Diseases

**DOI:** 10.1002/brb3.71269

**Published:** 2026-02-16

**Authors:** Ning Zhao, Shiheng Tan, Qingzhen Fu, Yanbing Li, Tian Tian, Zesong Cheng, Ding Zhang, Lijing Gao, Weiwei Bao, Depei Zhang, Zinan Li, Jinyin Liu, Liwan Wang, Zhuobo Zhang, Fan Wang, Yashuang Zhao

**Affiliations:** ^1^ Department of Epidemiology, School of Public Health Harbin Medical University Harbin P.R. China; ^2^ Department of Neurology The Fourth Affiliated Hospital of Harbin Medical University Harbin P.R. China; ^3^ NHC Key Laboratory of Etiology and Epidemiology (23618504) Harbin Medical University Harbin P.R. China

**Keywords:** GWAS, genetic association, "gut–brain axis," immune system

## Abstract

**Background:**

The “gut–brain axis” provides a theoretical foundation for the connection between intestinal and neurological diseases, but whether this reflects a shared genetic etiology or causal relationships exist remains unclear.

**Methods:**

We used genome‐wide association study summary data from FinnGen and UK Biobank to investigate the genetic correlations and causal relationships between three intestinal diseases and six neurological diseases.

**Results:**

We observed positive global genetic correlations between irritable bowel syndrome and epilepsy (*r*_g _= 0.429, *p *= 1.53 × 10^−2^), and stroke (*r*_g _= 0.368, *p *= 2.56×10^−2^). Upon dividing the whole genome into 1703 independent regions, local genetic correlations were identified in a region between ulcerative colitis and multiple sclerosis (Chr6: 31571218–32682664). We also identified 12 novel pleiotropic SNPs shared between intestinal and neurological diseases, as well as a functional gene shared between ulcerative colitis and multiple sclerosis. SNP heritability enrichment analysis indicated that ulcerative colitis and multiple sclerosis have enrichment in several immune cells. Two‐sample Mendelian randomization indicated the causal effect of Crohn's disease on Parkinson's disease (FDR = 1.34 × 10^−2^, OR = 1.092). The methylome Mendelian randomization analysis also showed causal relationships between several intestinal and neurological diseases.

**Conclusions:**

Through comprehensive and systematic statistical analysis, we identified the global and local genetic correlations and causal relationships between several intestinal and neurological diseases and discovered shared pleiotropic loci and genes between them. Furthermore, the consistent SNP heritability enrichment observed in immune cells also indicated the crucial role of the immune system in the “gut–brain axis.”

## Graphical abstract

We analyzed the genetic correlations and causal relationships between intestinal and neurological diseases, identifying global and local genetic associations between them. We identified shared pleiotropic loci and genes, as well as SNP heritability enrichments in tissues and cells. We also revealed potential causal relationships between these diseases from a multi‐omic perspective.

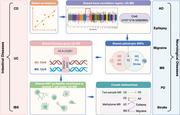

**Abbreviations**
AD: Alzheimer's disease
CD: Crohn's disease
CPASSOC: cross phenotype association
DALYs: disability‐adjusted life years
DNAm: DNA methylation
EWAS: epigenome‐wide association study
eQTL: expression quantitative trait loci
FDR: false discovery rate
GWAS: genome‐wide association study
HEIDI: heterogeneity in dependent instruments
IBD: inflammatory bowel disease
IBS: irritable bowel syndrome
IV: instrumental variable
IVW: inverse‐variance weighted
LDSC: linkage disequilibrium score regression
MHC: major histocompatibility complex
mQTL: metabolome quantitative trait loci
MR: Mendelian randomization
MS: multiple sclerosis
PD: Parkinson's disease
SMR: summary‐data‐based Mendelian randomization
SNP: single‐nucleotide polymorphism
UC: ulcerative colitis
ρ‐HESS: heritability estimation from summary statistics

## 1 Introduction

Inflammatory bowel disease (IBD) comprises Crohn's disease (CD) and ulcerative colitis (UC) (Abraham and Cho 2009), which are chronic and recurrent conditions with no permanent cure, leading to substantial long‐term morbidity (M'Koma 2013). In Western countries, the prevalence of IBD exceeds 0.3% (Ng et al. 2017), and irritable bowel syndrome (IBS) affects 7% to 21% of the general population (Chey et al. 2015). Neurological diseases, including stroke, Alzheimer's disease (AD), multiple sclerosis (MS), Parkinson's disease (PD), etc., are major causes of disability globally and are the second leading cause of death. The Global Burden of Disease study in 2019 revealed that stroke ranked third among the 369 diseases causing global disability‐adjusted life years (DALYs) (GBD 2019 Diseases and Injuries Collaborators 2020). Among neurological diseases causing DALYs, the top three are stroke (42.2%), migraine (16.3%), and AD (10.4%) (Feigin et al. 2020).

The “gut–brain axis” is considered a central mechanism mediating the association between gastrointestinal inflammation and neurodegenerative diseases, involving multiple complex regulatory pathways. Research indicates that in barrier tissues like the gut, neurons and immune cells form “neuro‐immune units” that enable bidirectional dynamic regulation (Veiga‐Fernandes and Artis 2018). Microbial metabolites, neurotransmitters, and hormones modulate glial cell function and the integrity of the blood–brain and gut barriers, thereby influencing the development and progression of AD and PD (Zhuang et al. 2024). Furthermore, the “indirect communication pathway” formed by intestinal lacteals and the meningeal lymphatic system not only transports microbial metabolites and immune cells but also plays a key role in clearing brain waste and relaying neuro‐immune signals (Loh et al. 2024). Deficiency or downregulation of the *Gpr35* receptor in intestinal epithelial cells may contribute to depression by affecting synapse integrity via microbial metabolites (Cheng et al. 2024). A shared genetic basis may underlie these “gut–brain” interactive mechanisms. Genome‐wide association studies (GWAS) have identified risk loci for both intestinal (Jostins et al. 2012; Liu et al. 2015; Sazonovs et al. 2022; Ek et al. 2015) and neurological diseases (Andrews et al. 2020; Nalls et al. 2019). Studies have demonstrated genetic association between IBD and PD (Kang et al. 2023), as well as between IBD and AD (Zeng et al. 2023). In addition to genetic variation in the genome, epigenetic variation also plays an important role in the development of diseases. Epigenome‐wide association studies (EWAS) have identified differential methylation sites associated with intestinal (Ventham et al. 2016) and neurological diseases (Chuang et al. 2017), but there is currently no research investigating the role of DNA methylation (DNAm) in the causal relationships between diseases including intestinal and neurological diseases. Two recent studies have conducted GWAS on specific gastrointestinal diseases and highlighted the need to systematically investigate shared genetic risks across different traits to advance our understanding of gut–brain interaction mechanisms (Yang et al. 2021; Witoelar et al. 2017). Therefore, integrating multi‐omics data and conducting a systematic analysis of the genetic associations between various intestinal and neurological diseases, to identify shared genetic architecture and possible causal relationships, is highly necessary.

In this study, we analyzed the global and local genetic correlations between these two types of diseases to understand the shared genetic basis and applied cross‐trait meta‐analysis to identify pleiotropic loci. Additionally, we utilized summary‐data‐based Mendelian randomization (SMR) to identify functional genes shared between these traits. To understand the SNP heritability specific to tissue and cell types shared between these diseases, we further examined the enrichment of SNP heritability in each disease. Notably, in exploring the causal relationships between intestinal and neurological diseases, we not only used traditional two‐sample Mendelian randomization (MR) but also integrated methylome summary data to investigate the causal effects of disease‐related CpG sites.

## 2 Materials and Methods

**FIGURE 1:**
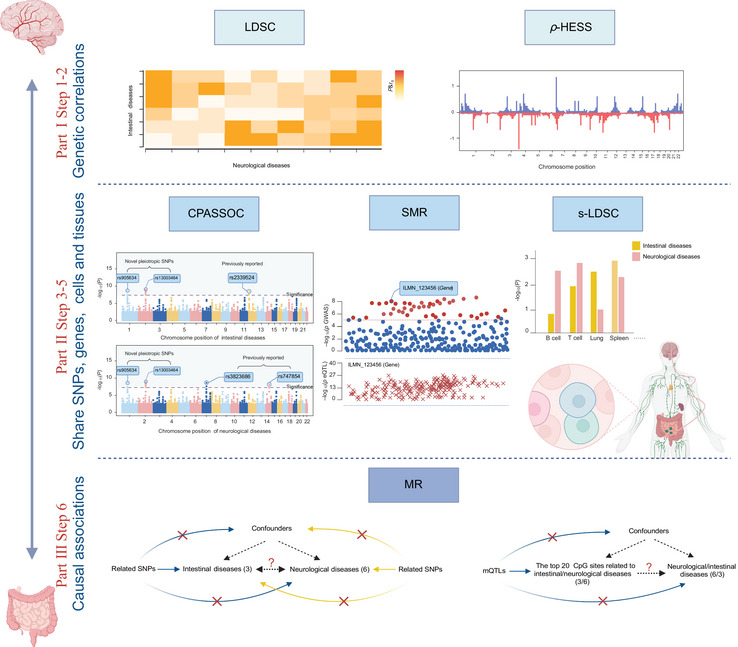
Overall study design of genome‐wide cross‐trait analysis. Our study employed various statistical analysis methods to investigate the genetic associations and causal relationships between three intestinal diseases and six neurological diseases. We utilized the following methods for analysis: LDSC (linkage disequilibrium score regression), global genetic correlation analysis; *ρ*‐HESS (heritability estimation from summary statistics), local genetic correlation analysis; CPASSOC (cross‐phenotype association), cross‐trait meta‐analysis; SMR (summary‐data‐based Mendelian randomization), Mendelian randomization analysis based on summary data; s‐LDSC (stratified‐linkage disequilibrium score regression), enrichment analysis of SNP heritability in tissues and cells; MR (Mendelian randomization), which identifies causal relationships.

The overall research design is shown in Figures 1 and S1.

### 2.1 Data Source

#### 2.1.1 GWAS Summary Data

GWAS summary data for intestinal diseases (CD, UC, and IBS) were obtained from the FinnGen database (Kurki et al. 2023), and data for neurological diseases (AD, epilepsy, migraine, MS, PD, and stroke) were obtained from the UK Biobank database (Karczewski et al. 2025). For the datasets not available in the UK Biobank, we used the Integrative Epidemiology Unit database (Hemani et al. 2018). The selection of these diseases was primarily based on their potential biological connections through the “gut–brain axis,” immune‐inflammatory pathways, and shared genetic mechanisms, as well as their high prevalence and substantial public health burden. For each trait, we selected the largest publicly available GWAS conducted in individuals of European ancestry with standardized quality control, in order to ensure sufficient statistical power and comparability across traits. Detailed characteristics of the datasets are presented in Table S1.

#### 2.1.2 eQTL Data

The eQTL (expression quantitative trait loci) summary‐level statistics were sourced from the Consortium for the Architecture of Gene Expression (Lloyd‐Jones et al. 2017), which includes five distinct cohorts, comprising 2765 individuals (predominantly Europeans), 38,624 gene expression probes, and 7,763,174 SNPs.

#### 2.1.3 DNAm and mQTL Data

DNAm data were obtained from the EWAS Catalog (Battram et al. 2022). We selected the top 20 CpG sites most correlated with each trait as exposures (Table S2). For each of these CpG sites, we derived mQTLs (methylation quantitative trait loci) robustly associated with their methylation level in whole blood from the Genetics of DNA Methylation Consortium (Min et al. 2021) as the instrumental variables (IVs).

#### 2.1.4 Tissue and Cell‐Type Specific Enrichment Data for SNP Heritability

We tested for SNP‐heritability enrichment of diseases in a set of 489 publicly available chromatin annotations. Of these annotations, 396 were from the Roadmap Epigenomics Consortium (Kundaje et al. 2015), and an additional 93 annotations were from the EN‐TEx projects (ENCODE Project Consortium 2012).

### 2.2 Statistical Analysis

#### 2.2.1 Global Genetic Correlation Analysis

To evaluate a shared genetic basis, we performed a global genetic correlation analysis using bivariate linkage disequilibrium score regression (LDSC) (Bulik‐Sullivan et al. 2015). LDSC utilized the slope from the regression of *z*‐scores on LD scores to estimate genetic correlation *r*_g_:

$$E\left[z_{1j}z_{2j}\right]=\frac{\sqrt{N_{1}N_{2}}\rho_{g}}{M}\;l_{j}+\frac{N_{s}\rho }{\sqrt{N_{1}N_{2}}}$$

$$r_{g}=\rho_{g}/\sqrt{h_{1}^{2}h_{2}^{2}}$$

For the specific explanation of the formula, please refer to the Supporting Information. A false discovery rate (FDR)‐corrected *p* value of 0.05 was used as the significance threshold (FDR *p *< 0.05).

#### 2.2.2 Local Genetic Correlation Analysis

The *ρ‐*heritability estimation from summary statistics (*ρ*‐HESS) (Shi et al. 2017) provided a precise measurement of the similarity between traits at specific regions in the genome, utilizing approximately 1703 independent LD blocks with an average length of 1.6 Mb (Berisa and Pickrell 2016). A Bonferroni correction was applied to adjust for multiple testing (two‐tailed *p *< 0.05/1703).

#### 2.2.3 Cross‐Trait Meta‐Analysis

We performed cross‐phenotype association (CPASSOC) (Zhu et al. 2015) to identify pleiotropic loci. CPASSOC used summary‐level data from single SNP‐trait associations from GWAS to identify variants associated with at least one trait. Significant SNPs were defined as index variants satisfying *p_Shet_* <5 × 10^−8^ and *p_single‐trait_* <1 × 10^−3^. If the SNPs were independent (LD *r* ^2 ^< 0.05 within 500‐kb windows) from genome‐wide significant SNPs in their respective single‐trait GWAS and satisfied *p_Shet_* <5 × 10^−8^ and 5×10^−8^ *<p_single‐trait_<*1 × 10^−3^, we then defined them as novel SNPs. We used 3DSNP (Lu et al. 2017) for detailed functional annotation of the identified pleiotropic SNPs.

#### 2.2.4 SMR analysis

We used SMR and HEIDI (heterogeneity in dependent instruments) test (Zhu et al. 2016) to identify potential functional genes underlying statistical associations between each disease pair. The approach used summary‐level data from GWAS and eQTL studies (Nica and Dermitzakis 2013) to test whether a transcript and phenotype are associated due to a shared causal variant (Wu et al. 2018). For eQTL data, we excluded probes located in the major histocompatibility complex (MHC) region and those with SNPs in the hybridization sequences; we retained 9538 probes for analysis.

#### 2.2.5 Tissue and Cell‐Type Specific Enrichment of SNP Heritability

To investigate the shared tissue and cell types between traits, we used the stratified‐LDSC (s‐LDSC) method to analyze tissue and cell enrichment (Finucane et al. 2015). For each tissue and cell in the dataset, we calculated the statistics for the differential expression of each gene. We then ranked genes based on their t‐statistics, took the top 10% of genes, and added 100 kb windows to obtain genomic annotation.

#### 2.2.6 MR Analysis

We used the R package “TwoSampleMR” (Hemani et al. 2018) to implement five different MR methods: MR‐Egger (Burgess and Thompson 2017), weighted median (Bowden et al. 2016), inverse variance weighted (IVW) (Burgess et al. 2015; Burgess et al. 2013), simple model, and weighted model (Hartwig et al. 2017). Causal estimates were considered significant if they were significant in the IVW method (FDR < 0.05) and remained direction‐consistent across the other four methods. To indicate the robustness of the MR results, we conducted several important sensitivity analyses, details of which can be found in the Supporting Information.

## 3 Results

### 3.1 Global Genetic Correlation

**FIGURE 2:**
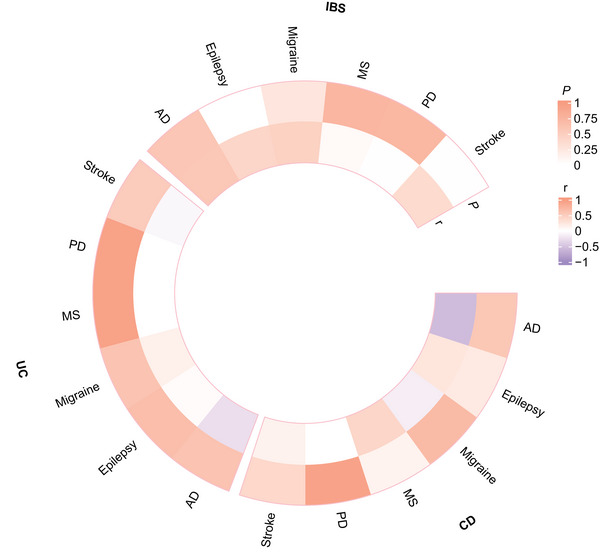
Circular heat map of genome‐wide genetic correlations between intestinal and neurological diseases. AD, Alzheimer's disease; CD, Crohn's disease; IBS, irritable bowel syndrome; MS, multiple sclerosis; PD, Parkinson's disease; UC, ulcerative colitis.

We used bivariate LDSC to estimate genetic correlations between intestinal diseases and neurological diseases. We found two significant positive genetic correlations between intestinal and neurological diseases, including IBS‐Epilepsy (*r_g _* = 0.429) and IBS‐Stroke (*r_g _* = 0.368) (Figure 2 and Table S3).

### 3.2 Local Genetic Correlation

We analyzed GWAS summary data of intestinal and neurological diseases to obtain local genetic correlations. We did not find significant local genetic correlations in the two pairs of traits with global genetic correlations. Among the joint traits without global genetic correlations, we found a significant local genetic signal between UC and MS in a region of chromosome 6 (31571218‐32682664), which is located in the MHC region (Figure S2 and Table S4).

### 3.3 Identification of Pleiotropic SNPs

**FIGURE 3:**
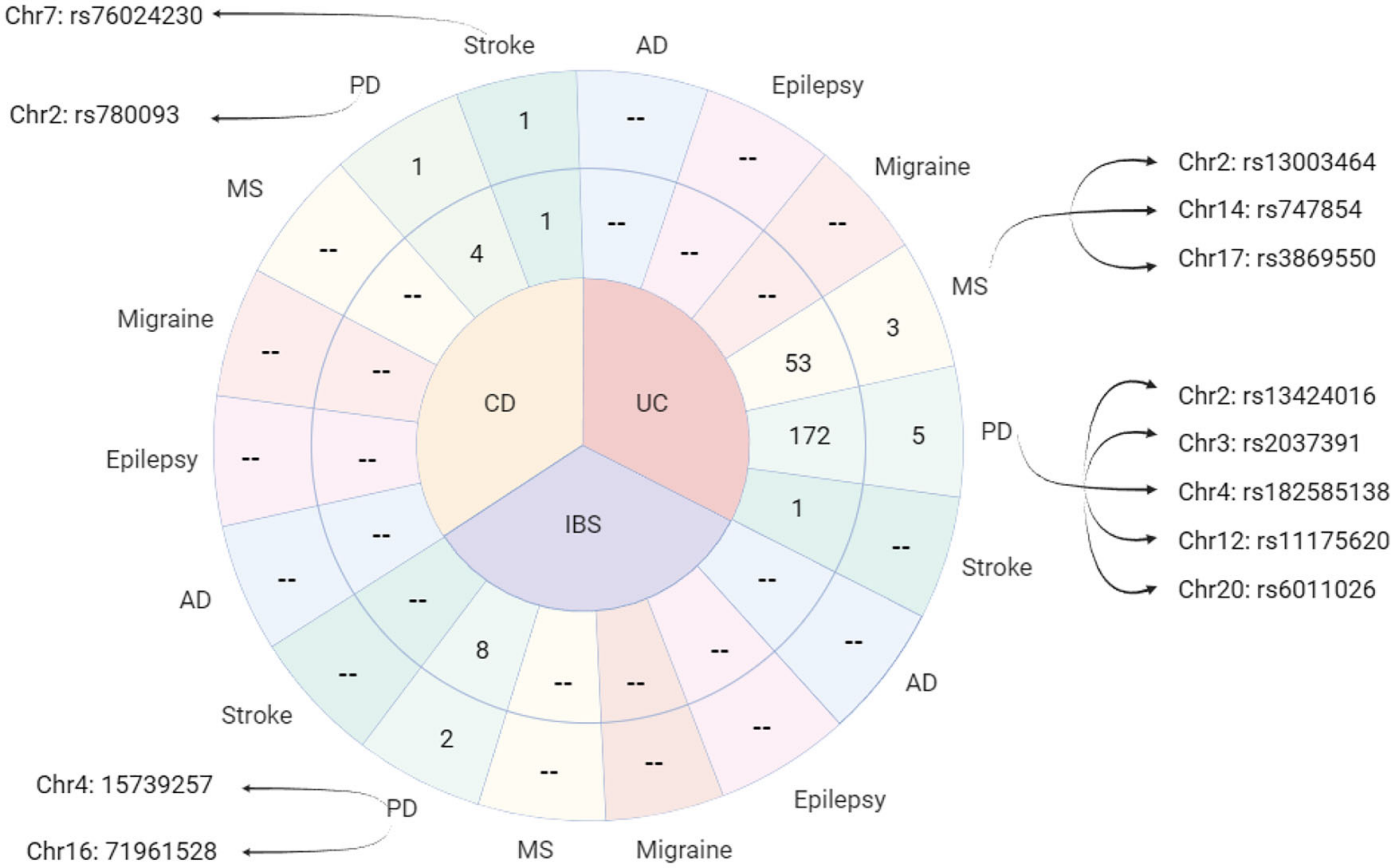
The shared SNP loci between intestinal and neurological diseases, excluding the MHC region. AD, Alzheimer's disease; CD, Crohn's disease; IBS, irritable bowel syndrome; MS, multiple sclerosis; PD, Parkinson's disease; UC, ulcerative colitis. The inner circle represents the number of SNPs exceeding the significance threshold, while the outer circle represents novel polygenic loci discovered through CPASSOC.

Based on the observed significant global genetic correlations between intestinal and neurological diseases, we performed cross‐trait meta‐analysis to identify pleiotropic loci between them. After excluding the MHC regions, 239 loci were identified at a genome‐wide significance level in CPASSOC (*p_Shet_* <5×10^−8^ and *p_single‐trait_* <1×10^−3^): CD‐PD (4), CD‐Stroke (1), UC‐PD (172), UC‐MS (53), UC‐Stroke (1), and IBS‐PD (8) (Table S5). After excluding 227 SNPs that were previously reported as single‐trait‐associated significant, we identified a total of 12 novel pleiotropic SNPs: one for CD and PD, one for CD and stroke; six for UC and PD, three for UC and MS; and two for IBS and PD (Figure 3 and Table S6). Additionally, we also identified 25 novel pleiotropic loci in the MHC region: CD‐MS (10), CD‐PD (2), UC‐MS (11), UC‐PD (1), IBS‐MS (1) (Table S7).

### 3.4 Identify the Shared Functional Genes

**FIGURE 4:**
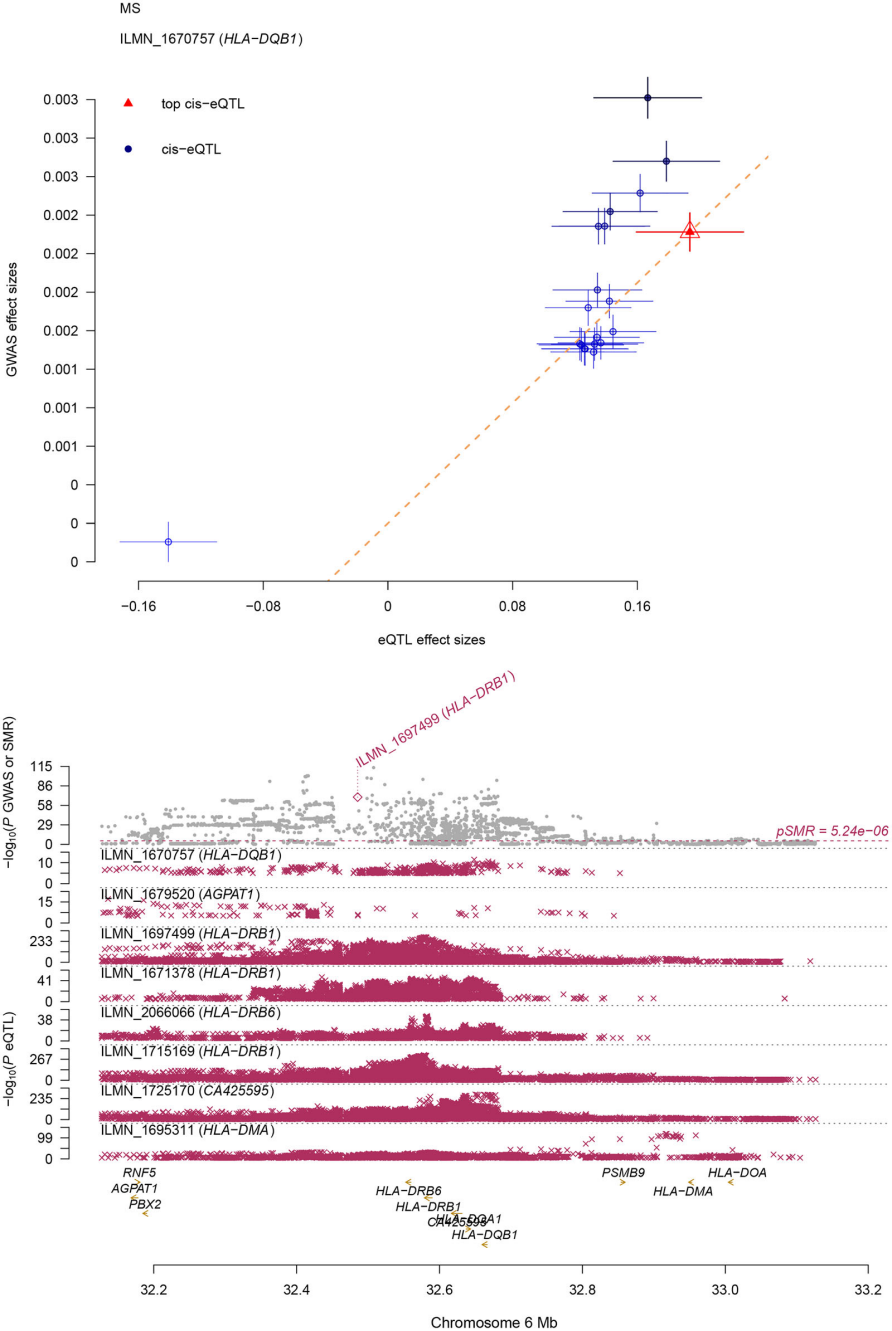
The shared gene between UC and MS. Prioritizing genes at a GWAS locus using SMR analysis. The results at the *HLA‐DQB1* locus for MS are shown. Top plot: The *p* values from the CAGE eQTL studies for *HLA‐DQB1* are displayed. All SNPs available in the GWAS and eQTL data are shown. The orange dashed lines represent the estimate of *b*_xy_ at the top cis‐eQTL (rather than the regression line). Error bars indicate the standard errors of SNP effects. Bottom plot, gray dots represent the *p* values for SNPs from GWAS, and diamonds represent the *p* values for probes from the SMR test. The gene *HLA‐DQB1*, highlighted in red, passed both the SMR and HEIDI tests.

We performed SMR and HEIDI tests for associations between gene expression and the 9 traits. We identified 47 genes (tagged by 58 gene expression probes) at a genome‐wide significance level (*p*_SMR _< 5.24 × 10^−6^) for CD, UC, MS, and PD (Table S8), of which 15 (tagged by 16 gene expression probes) survived the HEIDI‐outlier test (*p*_HEIDI_  > 0.01) (Table S9). Among these 15 genes, we found that *HLA‐DQB1* gene (tagged by *ILMN_1670757*) was shared by UC (*p*_SMR _= 7.89 × 10^−7^) and MS (*p*_SMR _= 1.19 × 10^−7^) (Figure 4 and Table S10).

### 3.5 Tissue and Cell‐Type Specific SNP Heritability Enrichment

Partitioning SNP heritability across nine diseases using data from 489 cell‐type‐specific annotations. We found significant enrichment of SNP heritability for UC and MS at FDR < 0.05. UC showed a significant specific SNP heritability enrichment in intestinal tissue. Next, we identified the tissues and cell‐types specific SNP heritability enrichment shared between UC and MS. At the cellular level, we observed significant SNP heritability enrichment for UC and MS in several T cell subtypes (Table S11).

### 3.6 Identify the Causal Relationships Between Diseases

#### 3.6.1 Bidirectional Two‐Sample MR

Next, we conducted MR analyses to investigate the consistency of the genetic overlap between intestinal and neurological diseases in terms of causal relationships. We discovered five MR methods to all 18 phenotypes pairs. We have discovered that genetic susceptibility to CD is associated with an increased risk of PD (FDR = 1.34 × 10^−2^, OR = 1.092) (Table S12). The sensitivity analysis indicated that there was no heterogeneity or horizontal pleiotropy in the causal relationship between the two variables. Furthermore, the research findings were not influenced by any outliers, demonstrating robustness (Figure S3).

#### 3.6.2 Methylome MR

**FIGURE 5:**
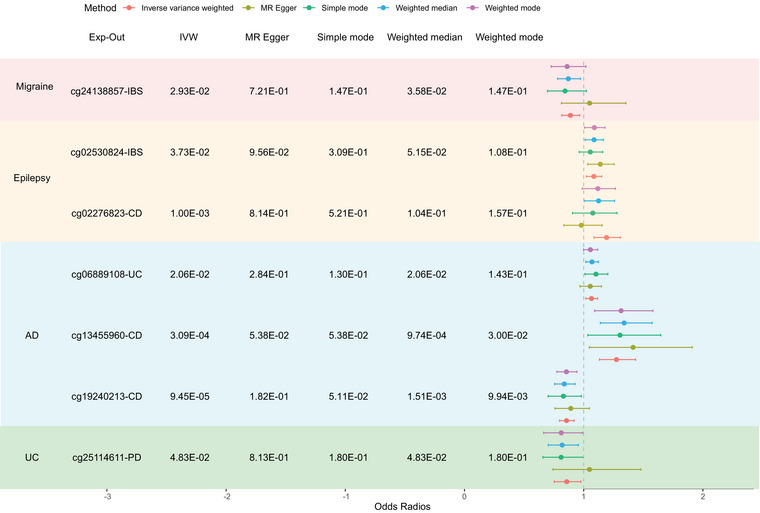
Summary of methylome MR analysis. AD, Alzheimer's disease; CD, Crohn's disease; IBS, irritable bowel syndrome; PD, Parkinson's disease; UC, ulcerative colitis. Error bars: the 95% confidence intervals (CIs) for the associated MR point estimates.

For methylome MR, we also performed bidirectional MR. From intestinal diseases to neurological diseases, we observed evidence of a causal effect between a pair of traits (FDR < 0.05), specifically UC‐related DNAm on PD at one CpG site (cg25114611, FDR = 4.83E‐02, OR = 0.857). For neurological diseases to intestinal diseases, we observed the causal effect between five trait pairs: AD‐related DNAm on CD at two CpG sites (cg13455960, FDR = 3.09E‐04, OR = 1.275; cg19240213, FDR = 9.45E‐05, OR = 0.856), AD‐related DNAm on UC at one CpG site (cg06889108, FDR = 2.06E‐02, OR = 1.065), epilepsy‐related DNAm on CD at one CpG site (cg02276823, FDR = 1.00E‐03, OR = 1.192), epilepsy‐related DNAm on IBS at one CpG site (cg02530824, FDR = 3.73E‐02, OR = 1.085), and migraine‐related DNAm on IBS at one CpG site (cg24138857, FDR = 2.93E‐02, OR = 0.888) (Figure 5 and Table S13).

## 4 Discussion

To our knowledge, this is the first study to comprehensively and systematically analyze the genetic associations and causal relationships between intestinal and neurological diseases using multi‐omics data.

Through LDSC analysis, we identified significant global genetic correlations between IBS and both epilepsy and stroke. Observational studies have also indicated a higher prevalence of IBS in epilepsy patients compared to the controls (Camara‐Lemarroy et al. 2016). Genetic associations between IBD and neurological diseases, particularly AD and PD, have been extensively studied (Kang et al. 2023; Adewuyi et al. 2022). However, our understanding of the genetic associations between IBS and neurological diseases remains limited. Our research, for the first time, from a genetic perspective, has revealed genome‐wide genetic associations between IBS and epilepsy as well as stroke.

However, whether the correlations were due to specific genomic regions or across the genome‐wide regions remains unclear. Furthermore, due to the mutual cancellation between positive and negative local heritability, there may exist local genetic correlations between traits even in the absence of global genetic associations. Therefore, we applied the *ρ*‐HESS to examine whether the genetic correlation between intestinal and neurological diseases is associated with specific genomic regions. We found a significant local genetic correlation between UC and MS in the MHC region (chromosome 6: 31571218–32682664). In addition, we identified a potential functional gene, *HLA‐DQB1*, shared by UC and MS within this region, along with 11 novel pleiotropic loci. Although this region's extensive linkage disequilibrium and polygenicity make it difficult to pinpoint causal variants, the finding is biologically plausible. Multiple studies have shown that MHC‐related genes are significantly associated with the onset and progression of IBD and MS (Lincoln et al. 2009; Heresbach et al. 1996; Cariappa et al. 1998; Stokkers et al. 1999; Ortiz et al. 2023). Previous studies have shown that polymorphisms in MHC‐II genes (*HLA‐DR*, *HLA‐DQ*) can reshape the gut microbiota and influence disease progression in MS mouse models (Shahi et al. 2021). This is consistent with the local genetic correlation between UC and MS that we observed in the MHC region. The shared MHC genetic background may alter gut microbial composition and impair intestinal barrier function, allowing pro‐inflammatory cytokines and microbial metabolites to enter the peripheral circulation and act on the central nervous system. This potential mechanism also suggests cross‐disease therapeutic opportunities; for example, targeting MHC‐related microbial dysbiosis or modulating pro‐inflammatory metabolites may help alleviate inflammation in both conditions. Future studies using independent cohorts, fine‐mapping analyses, and functional experiments are needed to further clarify the specific genes and underlying mechanisms.

We identified 12 novel loci shared between intestinal and neurological diseases, which are primarily located in these genes (*GCKR*, *ACTB*, *TMEM163*, *RTP3*, *LOC105377329*, *LRRK2*, *RTEL1*, *PUS10*, *ZFP36L1*, *STAT3*, *BST1*, *PKD1L3*). Studies have shown that common variants in the *GCKR* gene are not only closely associated with elevated triglycerides and glucose metabolism abnormalities (Sparsø et al. 2008), but also significantly correlate with systemic inflammatory markers, suggesting that *GCKR* may influence inflammation through metabolic regulation (Ligthart et al. 2016). Moreover, metabolic dysregulation and inflammation have been shown to alter gut microbiota composition and impair intestinal barrier function, providing a biological basis for the potential role of *GCKR* in the “gut–brain axis” (Rohr et al. 2020). The *LRRK2* gene is highly expressed in intestinal macrophages and dendritic cells, where it regulates gut immune responses and microbial homeostasis; knockout of *LRRK2* markedly alleviates intestinal inflammation and restores microbial balance (Yan et al. 2022). Moreover, *LRRK2* mutations are the most common pathogenic variants in PD, contributing to α‐synuclein aggregation, neuroinflammation, and lysosomal dysfunction. Studies have shown that increased *LRRK2* activity in the gut can activate central nervous system inflammation via the immune system (Cabezudo et al. 2023; Yang et al. 2025), providing direct molecular evidence for “gut–brain” interactions and suggesting that this gene may play a key role in the comorbidity and pathological mechanisms of UC and PD. The *STAT3* gene, as a central hub of immune and neuroinflammatory regulation, can trigger an imbalance in the intestinal *IL‐6/IL‐23/Th17* signaling axis when aberrantly activated, leading to mucosal barrier disruption and the release of pro‐inflammatory cytokines and microbial metabolites into the circulation (Jostins et al. 2012). These circulating inflammatory signals, together with *STAT3* activation in peripheral myeloid cells, can further act on the central nervous system, promoting T cell differentiation and glial cell activation, ultimately driving neuroinflammation and demyelination (Lu et al. 2020). This *STAT3*‐mediated “gut barrier disruption–systemic inflammation–neuroinflammatory activation” cascade provides a key molecular basis for the comorbidity of inflammatory bowel and neurological disease.

We observed consistent enrichment patterns of cell‐type‐specific heritability for UC and MS across multiple annotations, particularly in several T cell subsets (T helper cells, T helper 17 cells, and T helper memory cells). As autoimmune diseases, they are characterized by an exaggerated activation of T cells within the intestinal mucosa (Maul et al. 2005; Neurath 2014; Di Sabatino et al. 2012). The pathogenesis of UC is associated with various T helper cell subsets, including Th2, Th9, and Th17 (Zhang et al. 2020). A study has shown that injection of Th17 cells exacerbates the disease in mouse models of MS, while mice deficient in IL‐17 exhibit alleviated pathology (Komiyama et al. 2006). Therefore, appropriately modulating the immune response, particularly by inhibiting the differentiation and activation of Th17 cells, may represent effective strategies for the treatment of UC and MS.

So far, it is not clear whether there is a causal relationship between intestinal and neurological diseases. Some previous observational studies have indicated an association between these conditions. A national cohort study in Denmark showed a significant association between IBD and the later onset of PD (Villumsen et al. 2019). Unlike observational studies, our study used the two‐sample MR method to assess the causal relationship between intestinal and neurological diseases, which was not affected by confounding factors and reverse causality. Our research found that genetic susceptibility to CD does indeed increase the occurrence of PD. A study by Cui et al. (2022) also showed that genetically predicted PD was significantly associated with an increased risk of CD. However, Freuer and Meisinger (2022) showed that PD may not be directly caused by IBD, but by inflammatory mediators associated with the risk of PD, indicating no direct causal relationship between the two. This causal relationship suggests that intestinal inflammation may influence the development of PD by directly or indirectly affecting immune, neural, and microbial metabolic pathways along the “gut–brain axis.” Therefore, future studies with in‐depth experimental investigations are needed to elucidate the functional mechanisms and causal direction between them.

In addition to genomic variation, epigenetic variation may also play a role in the causal relationship between intestinal and neurological diseases. DNAm at the UC‐associated CpG site cg25114611 (*FKBP5*) decreases the risk of PD. Studies have shown that *FKBP5*, a key gene involved in stress response and HPA axis regulation, exhibits DNA methylation in peripheral blood that is associated with functional differences in the prefrontal–limbic circuitry (Kremer et al. 2024). This suggests that intestinal inflammation may influence the brain's susceptibility to neurodegenerative diseases by altering the epigenetic status of *FKBP5*. Our research also found that DNAm of the AD‐related CpG site cg13455960 (*RGMa*) increases the risk of CD occurrence. As an immune regulatory molecule, *RGMa* can promote T cell adhesion and inflammatory infiltration through the *Neogenin‐1/Rap1* signaling axis, thereby enhancing intestinal immune responses and contributing to the development of CD. This finding reveals, at the epigenetic level, the interaction between the gut and the brain, providing new molecular evidence for understanding bidirectional gut–brain regulation (Muramatsu et al. 2011).

Several limitations should be acknowledged. First, the genetic data used in this study were primarily derived from individuals of European ancestry, which may limit the generalizability of our findings to other populations. Second, the CpG sites were constrained by currently available large‐scale mQTL datasets, introducing potential selection bias. Our evaluation of tissue/cell‐type–specific heritability relied on the top 10% of most specific genes, which may overlook genes with weaker but potentially important effects. Additionally, the mQTL data were generated from peripheral blood, and DNAm patterns in blood may not fully reflect those in gut or brain tissues. Although we applied multiple approaches to mitigate bias arising from horizontal pleiotropy and incomplete colocalization, residual pleiotropy cannot be completely ruled out. Given the complex linkage disequilibrium structure and extreme polygenicity of the MHC region, findings from this region should be interpreted with caution and validated using independent datasets. Finally, multi‐omic integration is inherently constrained by the underlying data; differences in sample sizes across GWAS and QTL datasets may influence the precision of estimates and the statistical power of our analyses.

In conclusion, we provided evidence for the connection between the “gut–brain axis” from a genetic perspective. Specifically, in terms of genetic associations, we demonstrated the global and local genetic associations between several intestinal and neurological diseases, and identified pleiotropic loci and genes shared between them. Moreover, UC and MS have significant enrichment of SNP heritability in immune cells, underscoring the crucial role of the immune system in the “gut–brain axis.” In terms of causal relationships, we demonstrated the role of DNAm in the causal relationships of intestinal and neurological diseases, providing novel epigenetic insights into the mechanisms of the “gut–brain axis.”

## Author Contributions

Conceptualization: Yashuang Zhao, Ning Zhao, and Shiheng Tan. Data curation: Ning Zhao, Qingzhen Fu, and Yanbing Li. Formal analysis: Ning Zhao and Shiheng Tan. Funding acquisition: Yashuang Zhao. Investigation: Tian Tian and Zesong Cheng. Methodology: Ning Zhao, Ding Zhang, and Lijing Gao. Project administration: Yashuang Zhao and Fan Wang. Resources: Zhuobo Zhang. Software: Ning Zhao, Shiheng Tan, and Zinan Li. Supervision: Yashuang Zhao, Fan Wang, and Zhuobo Zhang. Validation: Weiwei Bao, Depei Zhang, Jinyin Liu, and Liwan Wang. Visualization: Ning Zhao, Shiheng Tan, and Qingzhen Fu. Writing – original draft: Ning Zhao. Writing – review and editing: Ning Zhao, Shiheng Tan, Yashuang Zhao, and Fan Wang.

## Funding

The authors have nothing to report.

## Ethics Statement

This study was a secondary analysis of the pooled data of GWAS, and therefore, no ethical review was required.

## Supporting information




**Supporting Information**: brb371269‐sup‐0001‐FigureS1.pdf

**Supporting Information**: brb371269‐sup‐0002‐SupMat.docx

## Data Availability

The GWAS summary data of neurological diseases and intestinal disease from FinnGen, UK Biobank, and Integrative Epidemiology Unit: FinnGen: an expedition into genomics and medicine | FinnGen. Pan UKBB | Pan UKBB (broadinstitute.org). http://gwas.mrcieu.ac.uk. The eQTL data are available from: http://cnsgenomics.com/software/smr/#DataResource. The DNAm and mQTL data are available from: MRC‐IEU EWAS Catalog, GoDMC Database. The tissue and cell‐type chromatin data are available from: Cell type specific analyses · bulik/ldsc Wiki · GitHub. LDSC: GitHub ‐ bulik/ldsc: LD Score Regression (LDSC). ρ‐HESS: https://huwenboshi.github.io/hess. CPASSOC: http://hal.case.edu/~xxz10/zhu‐web/. 3DSNP: https://www.omic.tech/3dsnp/. SMR: http://cnsgenomics.com/software/smr. S‐LDCS: https://github.com/bulik/ldsc/wiki/Cell‐type‐specific‐analyses. TwoSampleMR: https://mrcieu.github.io/TwoSampleMR/.
