# Supplementary material for: Unveiling Shared Genetic Architectures and Causality: Intestinal Diseases and Neurological Diseases
Source: Brain Behav. 2026 Feb 16;16(2):e71269. doi: 10.1002/brb3.71269 (PMC12910125; doi:10.1002/brb3.71269)
Supplement: Supplementary file 1 — Supporting Information: brb371269‐sup‐0001‐FigureS1.pdf [file BRB3-16-e71269-s002.pdf]

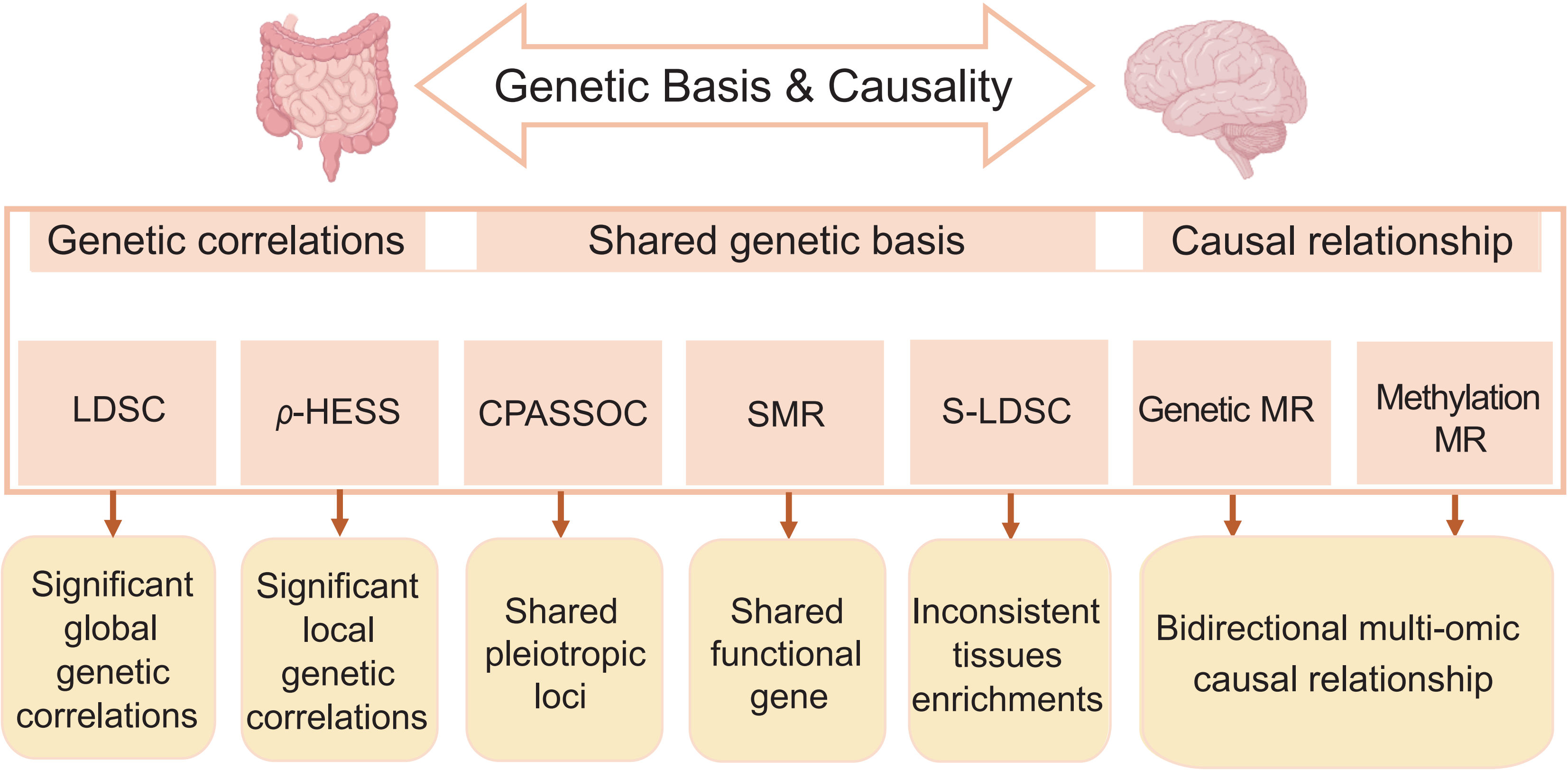
Genetic Basis & Causality
Genetic correlations
Shared genetic basis
Causal relationship
LDSC
ρ-HESS
CPASSOC
SMR
S-LDSC
Genetic MR
Methylation MR
Significant global genetic correlations
Significant local genetic correlations
Shared pleiotropic loci
Shared functional gene
Inconsistent tissues enrichments
Bidirectional multi-omic causal relationship
