# Supplementary material for: Unveiling Shared Genetic Architectures and Causality: Intestinal Diseases and Neurological Diseases
Source: Brain Behav. 2026 Feb 16;16(2):e71269. doi: 10.1002/brb3.71269 (PMC12910125; doi:10.1002/brb3.71269)
Supplement: Supplementary file 2 — Supporting Information: brb371269‐sup‐0002‐SupMat.docx [file BRB3-16-e71269-s001.docx]

**Supplementary Online Content**

**Unveiling Shared Genetic Architectures and Causality: Intestinal Diseases and Neurological Diseases**

**Author Information**

Ning Zhao^1^, Shiheng Tan^1^, Qingzhen Fu^1^, Yanbing Li^1^, Tian Tian^1^, Zesong Cheng^1^, Ding Zhang^1^, Lijing Gao^1^, Weiwei Bao^1^, Depei Zhang^1^, Zinan Li^1^, Jinyin Liu^1^, Liwan Wang^1^, Zhuobo Zhang^3*^, Fan Wang^1,2*^, Yashuang Zhao^1,2*^

**^*^Corresponding author:**

Yashuang Zhao, PhD, Department of Epidemiology, School of Public Health, Harbin Medical University, 157 Baojian Road, Harbin 150081, Heilongjiang Province, P.R. China. Email: zhao_yashuang@263.net. Tel: 86-(0)451-87502823

Fan Wang, PhD, Department of Epidemiology, School of Public Health, Harbin Medical University, 157 Baojian Road, Harbin 150081, Heilongjiang Province, P.R. China. Email: yifan.701@163.com. Tel: 86-(0)451-87502960

Zhuobo Zhang, PhD, Department of Neurology, The Fourth Affiliated Hospital of Harbin Medical University, 37 Yiyuan Street, Harbin 150006, Heilongjiang Province, P.R. China. Email: zzbzzx@hrbmu.edu.cn. Tel: 86-(0)451-82576950

**eMethods.** Study analyses

**Supplementary Table 1.** Basic characteristics of each GWAS summary data

**Supplementary Table 2.** The mQTL of the CpGs used for the MR analysis

**Supplementary Table 3.** Global genetic correlations between intestinal and neurological diseases

**Supplementary Table 4.** Local genetic correlation between intestinal and neurological diseases

**Supplementary Table 5.** Significant pleiotropic loci between intestinal and neurological diseases

**Supplementary Table 6.** Novel pleiotropic loci between intestinal and neurological diseases in the non-MHC region

**Supplementary Table 7.** Novel pleiotropic loci between intestinal and neurological diseases in the MHC region

**Supplementary Table 8.** Summary of functional genes for CD, UC, PD, and MS from SMR

**Supplementary Table 9.** Summary of functional genes for CD, UC, PD, and MS after HEIDE test

**Supplementary Table 10.** Functional genes shared between intestinal and neurological disease based on SMR

**Supplementary Table 11.** Summary of SNP heritability enrichment among for chromatin data

**Supplementary Table 12.** Causal relationship between CD and PD based on MR methods

**Supplementary Table 13.** Causal relationship between trait-associated CpG loci and diseases based on MR methods

**Supplementary Figure1.** Overall study design.

**Supplementary Figure2.** Local genetic correlation between intestinal and neurological diseases.

**Supplementary Figure3.** Sensitivity analysis of MR results.

**eMethods, Study Analyses**

**Data for DNAm**

First, we downloaded trait-associated datasets from the EWAS Catalog; for each trait, we took the intersection of all available datasets and ranked the CpG sites by ascending *p*-values, ultimately selecting the top 20 most significantly associated CpG sites for downstream analyses.

**Data for SMR analysis**

The expression quantitative trait loci (eQTL) summary-level statistics were obtained from the Consortium for the Architecture of Gene Expression (CAGE)^1^ study. The CAGE dataset consists of five distinct cohorts: BSGS (n=916)^2^, CAD (n=147)^3^, CHDWB (n=449)^4^, EGCUT (n=1,065)^5^, and Morocco (n=188)^6^, including a total of 2765 individuals (predominantly Europeans), 38,624 normalized gene expression probes, and 7,763,174 SNPs. Gene expression levels in these cohorts were measured in peripheral blood. Gene expression in this dataset was primarily measured using the Illumina HumanHT-12 v3.0 chip, with annotations based on hg19. We selected the probes with at least one cis-eQTL passing the significance threshold (*p*_eQTL_<5×10^−8^) and removed probes with SNPs in the hybridization sequences. After a series of quality controls, probes at the major histocompatibility complex (MHC) region and with SNPs in the hybridization sequences were excluded^7^. Finally, we retained 9,538 gene expression probes from CAGE for analysis.

**Data for tissue and cell-type specific enrichment data for SNP heritability**

We tested for SNP-heritability enrichment of diseases in a set of 489 publicly available tissue or cell-type-specific chromatin annotations. Of these, 396 annotations were originally created from five activating histone marks (H3K27ac, H3K4me3, H3K4me1, H3K9ac, and H3K36me3) and DNase I hypersensitivity regions, which were present in a subset of 88 tissues and cell types in the Roadmap Epigenomics Consortium^8^. An additional 93 annotations were created from a set of four activating histone marks (H3K27ac, H3K4me3, H3K4me1, and H3K36me3) in 27 tissues from EN‐TEx projects^9^.

**Statistical analyses**

**Global genetic correlation analysis**

LDSC^10^ relies on the fact that the GWAS effect-size estimate for a given SNP incorporates the effects of all SNPs in LD with that SNP. For a polygenic trait, SNPs with high LD will have higher χ^2^ statistics on average than SNPs with low LD. A similar relationship holds if we replace χ^2^ statistics for a single study with the product of z-scores from two studies of traits with non-zero genetic correlation. By implementing the algorithms described below, LDSC uses the slope from the regression of z-scores on LD-score to estimate *r*_g_ (the proportion of genetic variance shared by two traits divided by the square root of the product of their SNP heritability estimates, ranging from -1 to 1):

$$E[z_{1j}z_{2j}]=\frac{\sqrt{N_{1}N_{2}}\rho_{g}}{M}l_{j}+\frac{N_{s}\rho}{\sqrt{N_{1}N_{2}}}$$

$$r_{g}={\rho_{g}}/{\sqrt{h_{1}^{2}h_{2}^{2}}}$$

Here, *z_1j_* and *z_2j_* represent the z-scores of SNP_j_ from trait 1 and trait 2, respectively. *N_1_* and *N_2_* are the sample sizes for trait 1 and trait 2, *ρ_g_* is the genetic covariance, *l_j_* is the LD-score, *M* is the number of SNPs, *N_s_* is the number of overlapping samples, *ρ* is the phenotypic correlation in overlapping samples, and $h_{1}^{2}$ and $h_{2}^{2}$ are the SNP heritability of trait 1 and trait 2. LDSC analysis was performed using the known LD structure of European ancestry reference data from 1000 Genomes Project (1KGP) and was restricted to only HapMap3 SNPs, which are recognized as well imputed in most studies to minimize bias due to low imputation quality. In addition, LDSC intercepts can be used to detect sample overlap. To avoid duplicate samples between exposure and outcome datasets, we strictly controlled the data sources for intestinal and neurological diseases. For datasets confirmed to have overlapping samples, we validated the results using alternative datasets from different sources to ensure their reliability.

**Estimation of local genetic correlations using *ρ*-HESS**

We estimated local SNP heritability and genetic covariance across traits using *ρ*-HESS^11^, and then calculated local genetic correlation estimates based on local single-trait SNP heritability and local cross-trait genetic covariance estimates.

**Cross-trait meta-analysis**

CPASSOC^12^ provides two statistics, *S_Hom_* and *S_Het_*. *S_Hom_* is similar to the fixed effect meta-analysis method but accounts for the correlations of summary statistics among traits and among cohorts induced by correlated traits, potential overlapping or related samples. However, it is less powerful under the presence of between-study heterogeneity. As an extension of *S_Hom_*, *S_Het_* maintains statistical power even in the presence of heterogeneity by assigning more weight to the larger trait-specific effect sizes. Therefore, this method (*S_Het_*) was adopted for the analysis.

We obtained independent loci using the ‘clump-data’ function in the R package ‘TwoSampleMR’^13^. If they were independent (LD r^2^<0.05 within 500-kb windows) from genome-wide significant SNPs in their respective single-trait GWAS and satisfied *p_Shet_*<5×10^-8^ and 5×10^-8^*<p_single-trait_<*1×10^-3^, we defined them as the novel SNPs. We used 3DSNP^14^ for detailed functional annotation of the identified pleiotropic SNPs.

**Summary-data-based Mendelian randomization**

Compared with most other methods for integrative analysis of GWAS and eQTL data^15-17^, the SMR & HEIDI approach can distinguish between a pleiotropic model (where gene expression and phenotype are associated due to a single shared genetic variant) and a linkage model (where two or more distant genetic variants in LD independently affect gene expression and phenotype)^18^. Moreover, like other summary-data-based methods, the SMR & HEIDI approach allows the use of GWAS and eQTL data from two independent studies. Thus, the statistical power can be increased by using data from studies with very large sample sizes.

**Mendelian randomization analysis**

We used five different MR methods to explore potential causal relationships, and each method was analyzed using bidirectional MR. For each method, independent SNPs with genome-wide association evidence (*p*≤5×10^−8^) for the ‘exposure’ trait were used as instrumental variables (IVs). We excluded SNPs with r^2^>0.01 within 10000kb and merged the remaining SNPs with those from the ‘outcome’ trait. We calculated the F-statistic for each SNP to measure its strength as an IV. SNPs with the *F*<10, considered as weak IVs, were excluded. Causal estimates were considered significant if they were significant in the IVW method (FDR<0.05) and remained directionally consistent across the other four methods.

For the methylome MR, we selected mQTL with a *p*<1×10^-8^ for cis-mQTL (SNP<1 Mb from CpG site) and a *p*<1×10^-14^ for trans-mQTL (defined as distal variants>1 Mb from the CpG site), FDR<0.05 was considered significant.

The sensitivity analyses included the following steps. First, we performed a heterogeneity test to examine the differences between the individual IVs. If *p*<0.05, indicating heterogeneity among the SNPs, a random effects model was used. Second, we applied the MR-Egger regression method to test for horizontal pleiotropy using the intercept. If the intercept differed significantly from zero, it suggested the presence of horizontal pleiotropy. Finally, we performed the leave-one-out analysis, where one SNP was removed at a time and MR was conducted based on the remaining SNPs.

**Data availability:**

The GWAS summary data of neurological diseases and intestinal disease from FinnGen, UK Biobank, and [Integrative Epidemiology Unit](https://bris.ac.uk/ieu):

[FinnGen: an expedition into genomics and medicine | FinnGen](https://www.finngen.fi/en)

[Pan UKBB | Pan UKBB (broadinstitute.org)](https://pan.ukbb.broadinstitute.org/)

<http://gwas.mrcieu.ac.uk>.

The eQTL data are available from:

<http://cnsgenomics.com/software/smr/#DataResource>.

The DNAm and mQTL data are available from:

[MRC-IEU EWAS Catalog](https://ewascatalog.org/)

[GoDMC Database](http://mqtldb.godmc.org.uk/index.php)

The tissue and cell-type chromatin data are available from:

[Cell type specific analyses · bulik/ldsc Wiki · GitHub](https://github.com/bulik/ldsc/wiki/Cell-type-specific-analyses).

**Code availability:**

LDSC: [GitHub - bulik/ldsc: LD Score Regression (LDSC)](https://github.com/bulik/ldsc)

ρ-HESS: <https://huwenboshi.github.io/hess>

CPASSOC: <http://hal.case.edu/~xxz10/zhu-web/>

3DSNP: <https://www.omic.tech/3dsnp/>

SMR: <http://cnsgenomics.com/software/smr>

S-LDCS: <https://github.com/bulik/ldsc/wiki/Cell-type-specific-analyses>

TwoSampleMR: <https://mrcieu.github.io/TwoSampleMR/>

**Supplementary Table 1. Basic characteristics of each GWAS summary data.**

| **Trait** | **Ncase** | **Ncontrol** | **Population** | **Source** |
| --- | --- | --- | --- | --- |
| CD | 2033 | 409940 | European | Finngen |
| UC | 5931 | 405386 | European | Finngen |
| IBS | 10329 | 329381 | European | Finngen |
| AD | 954 | 487331 | European | / |
| Epilepsy | 2326 | 460684 | European | UKB |
| Migraine | 1001 | 462009 | European | UKB |
| MS | 1679 | 461254 | European | UKB |
| PD | 33674 | 449056 | European | IPDGC |
| Stroke | 6116 | 456817 | European | UKB |

Trait represents the three categories of intestinal disease and six categories of neurological diseases included in this study**.** All the GWAS study populations included in this study are of European descent. Ncase and Ncontrol: the sample size of cases and controls in each GWAS; Source: the origin of each GWAS study; CD: Crohn's disease; UC: ulcerative colitis; IBS: irritable bowel syndrome; AD: Alzheimer's disease; MS: multiple sclerosis; PD: Parkinson's disease; IPDGC: International Parkinson's Disease Genomics Consortium.

**Supplementary Table 2. The top 20 CpG sites most associated with intestinal and neurological diseases.**

| **Trait** | **CpG** | **Chr** | **Position** | **Gene** | ***p*** |
| --- | --- | --- | --- | --- | --- |
| CD | cg26470501 | 19 | 45252955 | *BCL3* | 1.50E-16 |
|  | cg15551881 | 9 | 123688715 | *TRAF1* | 2.35E-16 |
|  | cg02508743 | 8 | 56903623 | *LYN* | 4.65E-15 |
|  | cg26599989 | 11 | 1297087 | *TOLLIP* | 5.42E-15 |
|  | cg16411857 | 16 | 57023191 | *NLRC5* | 1.88E-14 |
|  | cg02448796 | 1 | 6101339 | *KCNAB2* | 1.97E-14 |
|  | cg08289839 | 13 | 111318640 | *CARS2* | 1.54E-13 |
|  | cg17980786 | 3 | 32933637 | *TRIM71* | 2.08E-13 |
|  | cg13619623 | 7 | 33637324 | *BBS9* | 4.05E-13 |
|  | cg06192883 | 15 | 52554171 | *MYO5C* | 4.07E-13 |
|  | cg14722693 | 8 | 19436451 | *CSGALNACT1* | 7.55E-13 |
|  | cg11095027 | 11 | 1297066 | *TOLLIP* | 1.18E-12 |
|  | cg06946797 | 16 | 11422409 | *RMI2* | 1.96E-12 |
|  | cg22753611 | 6 | 17472892 | *CAP2* | 3.30E-12 |
|  | cg07148145 | 7 | 99437259 | *CYP3A43* | 3.85E-12 |
|  | cg23740758 | 6 | 11324433 | *NEDD9* | 5.16E-12 |
|  | cg24469729 | 7 | 27160520 | *HOXA3* | 9.58E-12 |
|  | cg00782811 | 6 | 46293734 | *RCAN2* | 1.11E-11 |
|  | cg00634542 | 2 | 219254588 | *SLC11A1* | 1.23E-11 |
|  | cg27243685 | 21 | 43642366 | *ABCG1* | 1.29E-11 |
| UC | cg25114611 | 6 | 35696870 | *FKBP5* | 3.86E-13 |
|  | cg01059398 | 3 | 172235808 | *TNFSF10* | 1.09E-12 |
|  | cg18608055 | 19 | 1130866 | *SBNO2* | 2.16E-12 |
|  | cg16936953 | 17 | 57915665 | *VMP1* | 3.73E-12 |
|  | cg17501210 | 6 | 166970252 | *RPS6KA2* | 4.05E-12 |
|  | cg26804423 | 7 | 8201134 | *ICA1* | 8.18E-12 |
|  | cg22959742 | 10 | 13913931 | *FRMD4A* | 3.76E-11 |
|  | cg12170787 | 19 | 1130965 | *SBNO2* | 8.11E-11 |
|  | cg13619623 | 7 | 33637324 | *BBS9* | 1.18E-10 |
|  | cg26227957 | 1 | 19547285 | *EMC1* | 1.43E-10 |
|  | cg20228731 | 7 | 130646051 | *FLJ43663* | 2.69E-10 |
|  | cg23729283 | 7 | 29040109 | *CPVL* | 2.84E-10 |
|  | cg16755922 | 17 | 80536214 | *FOXK2* | 3.20E-10 |
|  | cg25533551 | 19 | 48823178 | *ODAD1* | 3.21E-10 |
|  | cg18942579 | 17 | 57915773 | *VMP1* | 5.28E-10 |
|  | cg23761815 | 10 | 73083123 | *SLC29A3* | 6.45E-10 |
|  | cg27243685 | 21 | 43642366 | *ABCG1* | 6.66E-10 |
|  | cg26955383 | 10 | 105218660 | *CALHM1* | 7.32E-10 |
|  | cg15928106 | 7 | 130646078 | *FLJ43663* | 9.53E-10 |
|  | cg16724148 | 1 | 100326338 | *AGL* | 9.60E-10 |
| IBS | cg25210835 | 7 | 110254828 | *GSTM5* | 6.00E-03 |
|  | cg20803293 | 7 | 110254709 | *GSTM5* | 2.00E-03 |
|  | cg24467349 | 7 | 110254835 | *GSTM5* | 2.00E-03 |
|  | cg17751872 | 4 | 21264982 | *ZNF714* | 6.00E-03 |
|  | cg09352518 | 4 | 21265421 | *ZNF714* | 2.40E-02 |
|  | cg01483656 | 4 | 21264896 | *ZNF714* | 1.20E-02 |
|  | cg16653991 | 4 | 123244536 | *FGFR2* | 1.00E-02 |
|  | cg02210151 | 4 | 123356041 | *FGFR2* | 4.50E-02 |
|  | cg08899523 | 4 | 123244591 | *FGFR2* | 7.00E-03 |
|  | cg06791446 | 4 | 123355268 | *FGFR2* | 5.00E-03 |
|  | cg24506221 | 3 | 110230401 | *GSTM1* | 8.00E-03 |
|  | cg22879098 | 2 | 672845 | *TPPP* | 5.00E-03 |
|  | cg24082121 | 2 | 672872 | *TPPP* | 1.00E-02 |
|  | cg00867835 | 2 | 149484985 | *SSPO* | 3.00E-03 |
|  | cg01996567 | 2 | 149484880 | *SSPO* | 6.00E-03 |
|  | cg25537993 | 2 | 58545182 | *ZSCAN1* | 2.00E-02 |
|  | cg24368848 | 2 | 58545160 | *ZSCAN1* | 4.50E-02 |
|  | cg13697193 | 2 | 12849138 | *GPR19* | 0.00E+00 |
|  | cg03933279 | 2 | 12849136 | *GPR19* | 1.00E-03 |
|  | cg19867917 | 2 | 3642629 | *COLEC11* | 2.80E-02 |
| AD | cg01419713 | 8 | 42038135 | *PLAT* | 2.20E-14 |
|  | cg11823178 | 8 | 41519399 | *ANK1; MIR486* | 3.24E-13 |
|  | cg07061298 | 7 | 27153847 | *HOXA3* | 4.57E-13 |
|  | cg03223072 | 10 | 116398913 | *ABLIM1* | 1.10E-12 |
|  | cg05066959 | 8 | 41519308 | *ANK1; MIR486* | 1.45E-12 |
|  | cg19240213 | 7 | 27163095 | *HOXA3* | 2.29E-12 |
|  | cg10045881 | 1 | 111770291 | *CHI3L2* | 2.38E-12 |
|  | cg01111041 | 6 | 32121055 | *PPT2; PRRT1* | 6.83E-12 |
|  | cg16988611 | 10 | 82224946 | *TSPAN14* | 9.98E-12 |
|  | cg01681367 | 16 | 29676071 | *SPN* | 1.25E-11 |
|  | cg01301319 | 7 | 27153580 | *HOXA3* | 1.54E-11 |
|  | cg02317313 | 12 | 122235206 | *LOC338799* | 1.69E-11 |
|  | cg12234455 | 19 | 49220235 | *MAMSTR* | 3.07E-11 |
|  | cg21806242 | 11 | 72532891 | *ATG16L2* | 3.22E-11 |
|  | cg18757087 | 5 | 173317332 | *CPEB4* | 4.42E-11 |
|  | cg00621289 | 21 | 47855916 | *PCNT* | 4.69E-11 |
|  | cg06889108 | 5 | 173317342 | *CPEB4* | 5.00E-11 |
|  | cg03169557 | 16 | 89598950 | *SPG7* | 5.41E-11 |
|  | cg17104258 | 1 | 167090646 | *DUSP27* | 6.27E-11 |
|  | cg13455960 | 15 | 93617134 | *RGMA* | 6.93E-11 |
| Epilepsy | cg00044050 | 16 | 11439710 | *C16orf75* | 0 |
|  | cg00074086 | 7 | 4901785 | *PAPOLB; RADIL* | 0 |
|  | cg00259046 | 11 | 50257771 | *LOC441601* | 0 |
|  | cg00419321 | 1 | 75593476 | *LHX8* | 0 |
|  | cg00700969 | 10 | 4868386 | *AKR1E2* | 0 |
|  | cg00817731 | 3 | 46759475 | *PRSS50* | 0 |
|  | cg00976097 | 5 | 421733 | *AHRR* | 0 |
|  | cg01005080 | 1 | 75592950 | *LHX8* | 0 |
|  | cg01124132 | 22 | 32599511 | *RFPL2* | 0 |
|  | cg01175142 | 10 | 118387327 | *PNLIPRP2* | 0 |
|  | cg01559901 | 19 | 12624832 | *ZNF709* | 0 |
|  | cg01577475 | 2 | 114033581 | *PAX8; LOC440839* | 0 |
|  | cg01788113 | 3 | 46759472 | *PRSS50* | 0 |
|  | cg01820273 | 6 | 35754906 | *C6orf127* | 0 |
|  | cg01951420 | 3 | 121553821 | *IQCB1; EAF2* | 0 |
|  | cg01977519 | 19 | 7504058 | *ARHGEF18* | 0 |
|  | cg02138082 | 1 | 978573 | *AGRN* | 0 |
|  | cg02197629 | 21 | 15352848 | *C21orf81* | 0 |
|  | cg02276823 | 7 | 5000111 | *MMD2* | 0 |
|  | cg02530824 | 4 | 74847766 | *PF4* | 0 |
| Migraine | cg23519572 | 13 | 114808934 | *RASA3* | 1.20E-04 |
|  | cg11830876 | 17 | 78006238 | *TBC1D16* | 1.86E-05 |
|  | cg26549860 | 11 | 72554159 | *FCHSD2* | 1.49E-04 |
|  | cg14609458 | 11 | 32851177 | *PRRG4* | 1.32E-05 |
|  | cg11127249 | 10 | 6104630 | *IL2RA* | 1.63E-04 |
|  | cg21820923 | 15 | 83680418 | *C15orf40* | 1.21E-04 |
|  | cg20004324 | 12 | 24610099 | *SOX5* | 6.79E-05 |
|  | cg17919020 | 7 | 150755629 | *CDK5; SLC4A2* | 1.91E-05 |
|  | cg24569624 | 11 | 6280804 | *CCKBR* | 9.16E-05 |
|  | cg27396830 | 1 | 11760709 | *C1orf187* | 1.09E-07 |
|  | cg06486088 | 2 | 237010222 | *AGAP1* | 7.93E-05 |
|  | cg26820922 | 2 | 32503023 | *YIPF4* | 5.39E-05 |
|  | cg24136937 | 7 | 1012013 | *COX19* | 1.30E-04 |
|  | cg24138857 | 6 | 32055079 | *TNXB* | 7.68E-05 |
|  | cg16194540 | 10 | 131934790 | *GLRX3* | 5.41E-05 |
|  | cg16661609 | 19 | 55172983 | *LILRB4* | 1.11E-04 |
|  | cg06837034 | 4 | 79093019 | *FRAS1* | 6.18E-05 |
|  | cg22829706 | 6 | 133079987 | *VNN2* | 1.11E-04 |
|  | cg01683270 | 4 | 8136996 | *ABLIM2* | 1.24E-04 |
|  | cg24361350 | 1 | 6750722 | *DNAJC11* | 1.02E-04 |
| MS | cg26062856 | 15 | 26109425 | *ATP10A* | 1.50E-07 |
|  | cg00959259 | 3 | 122281975 | *PARP9; DTX3L* | 2.70E-07 |
|  | cg14870271 | 17 | 76976010 | *LGALS3BP* | 3.20E-07 |
|  | cg24876035 | 19 | 7682769 | *KIAA1543* | 4.80E-07 |
|  | cg06872964 | 1 | 79085250 | *IFI44L* | 6.30E-07 |
|  | cg04927537 | 17 | 76976091 | *LGALS3BP* | 1.10E-06 |
|  | cg22930808 | 3 | 122281881 | *PARP9; DTX3L* | 1.40E-06 |
|  | cg02727423 | 14 | 100258973 | *EML1* | 2.00E-06 |
|  | cg10552523 | 11 | 313478 | *IFITM1* | 2.80E-06 |
|  | cg22713958 | 17 | 76976245 | *LGALS3BP* | 3.40E-06 |
|  | cg17009297 | 5 | 158526642 | *EBF1* | 4.60E-06 |
|  | cg16492851 | 7 | 1062681 | *C7orf50; MIR339* | 5.70E-06 |
|  | cg12978253 | 10 | 134258602 | *C10orf91* | 5.90E-06 |
|  | cg06385584 | 7 | 148784696 | *ZNF786* | 7.20E-06 |
|  | cg17988326 | 1 | 41091703 | *RIMS3* | 8.20E-06 |
|  | cg19596541 | 7 | 32932066 | *KBTBD2* | 8.30E-06 |
|  | cg03736062 | 4 | 113208476 | *TIFA* | 9.40E-06 |
|  | cg07470694 | 15 | 26109249 | *ATP10A* | 9.60E-06 |
|  | cg08659179 | 5 | 27038707 | *CDH9* | 1.00E-05 |
|  | cg00397545 | 19 | 45417526 | *APOC1* | 1.20E-05 |
| PD | cg04772575 | 12 | 123431865 | *ABCB9* | 4.33E-10 |
|  | cg09993145 | 1 | 25291905 | *RUNX3* | 3.59E-09 |
|  | cg09032544 | 1 | 167487295 | *CD247* | 4.69E-09 |
|  | cg26341831 | 1 | 226036279 | *TMEM63A* | 5.21E-09 |
|  | cg21577598 | 17 | 80084751 | *CCDC57* | 8.11E-09 |
|  | cg26793227 | 1 | 16483658 | *EPHA2* | 8.17E-09 |
|  | cg16270399 | 18 | 74257894 | *LOC284276* | 8.95E-09 |
|  | cg22358291 | 4 | 10101553 | *WDR1* | 1.38E-08 |
|  | cg24339704 | 19 | 2529022 | *GNG7* | 2.32E-08 |
|  | cg02600394 | 4 | 48136234 | *TXK* | 2.44E-08 |
|  | cg23207054 | 17 | 38171530 | *CSF3* | 2.79E-08 |
|  | cg19081101 | 1 | 203156625 | *CHI3L1* | 3.05E-08 |
|  | cg17879101 | 10 | 126329354 | *FAM53B* | 3.37E-08 |
|  | cg26489413 | 11 | 10476976 | *AMPD3* | 4.15E-08 |
|  | cg20720686 | 7 | 75582881 | *POR* | 4.16E-08 |
|  | cg02861056 | 2 | 68592345 | *PLEK* | 4.86E-08 |
|  | cg13060970 | 2 | 68592349 | *PLEK* | 4.93E-08 |
|  | cg14642045 | 12 | 109538736 | *UNG* | 5.17E-08 |
|  | cg08400494 | 13 | 111318490 | *CARS2* | 5.34E-08 |
|  | cg16000989 | 4 | 41983716 | *DCAF4L1* | 5.54E-08 |
| Stroke | cg09238598 | 5 | 14871908 | *ANKH* | 7.30E-18 |
|  | cg09643587 | 3 | 107809710 | *CD47* | 1.68E-15 |
|  | cg04192862 | 10 | 28966472 | *BAMBI* | 2.85E-15 |
|  | cg10122474 | 14 | 88459471 | *GALC* | 2.16E-14 |
|  | cg06825833 | 13 | 111566802 | *ANKRD10* | 6.47E-14 |
|  | cg11424525 | 21 | 46494378 | *ADARB1* | 1.25E-13 |
|  | cg08308556 | 6 | 157342734 | *ARID1B* | 5.86E-13 |
|  | cg13470032 | 9 | 214612 | *C9orf66* | 9.05E-13 |
|  | cg06014792 | 17 | 7382760 | *ZBTB4* | 1.29E-12 |
|  | cg25506900 | 17 | 73512855 | *CASKIN2* | 5.70E-12 |
|  | cg07806715 | 19 | 48018254 | *NAPA* | 7.32E-12 |
|  | cg05425664 | 17 | 57184151 | *TRIM37* | 7.35E-12 |
|  | cg16573386 | 1 | 1334508 | *CCNL2* | 9.97E-12 |
|  | cg17116739 | 16 | 67063591 | *CBFB* | 1.34E-11 |
|  | cg06465196 | 4 | 154387603 | *KIAA0922* | 1.68E-11 |
|  | cg06202984 | 9 | 98079903 | *FANCC* | 6.63E-11 |
|  | cg21475747 | 5 | 177631425 | *HNRNPAB* | 8.63E-11 |
|  | cg09567732 | 5 | 177631413 | *HNRNPAB* | 1.03E-10 |
|  | cg08522087 | 5 | 14871910 | *ANKH* | 1.26E-10 |
|  | cg18644710 | 2 | 201172048 | *SPATS2L* | 5.80E-10 |

CD: Crohn's disease; UC: ulcerative colitis; IBS: irritable bowel syndrome; AD: Alzheimer's disease; MS: multiple sclerosis; PD: Parkinson's disease; Chr: chromosome.**Supplementary Table 3. Global genetic correlations between intestinal and neurological diseases.**

| **Trait 1** | **Trait 2** | ***r_g_*** | **SE** | ***p*** |
| --- | --- | --- | --- | --- |
| IBS | Epilepsy  Stroke | 0.429  0.368 | 0.177  0.165 | 1.53E-02  2.56E-02 |

IBS: irritable bowel syndrome; *r_g_*, genetic correlation; SE, standard error.

**Supplementary table 4. Local genetic correlation for intestinal and neurological diseases.**

| **Traits** | **Chr: Position** | **No. SNPs** | **Local_rhog** | **Variance** | **SE** | **Z-socre** | ***p*** |
| --- | --- | --- | --- | --- | --- | --- | --- |
| UC-MS | chr6: 31571218-32682664 (**MHC region**) | 2015 | 6.05E-04 | 9.73E-09 | 9.86E-05 | 6.135 | 8.50E-10 |

Chr: chromosome; Position: independent LD regions on chromosome; No. SNP: number of SNPs in each region; Local_rhog: the size of the local genetic correlation statistic; SE: standard error; UC: ulcerative colitis; MS: multiple sclerosis.

**Supplementary table 5. Significant pleiotropic loci between intestinal and neurological diseases (*P*_CPASSOC_ < 5×10^-8^, *P*_single-trait_< 1×10^-3^).**

| **Traits** | **SNP** | **Chr** | **Position** | **A1** | **A2** | **b_1** | **SE_1** | ***P*_1** | **b_2** | **SE_2** | ***P*_2** | ***P*_Shet** |
| --- | --- | --- | --- | --- | --- | --- | --- | --- | --- | --- | --- | --- |
| CD-PD | rs4665972 | 2 | 27375230 | C | T | -0.157 | 0.032 | 8.72E-07 | 0.071 | 0.018 | 7.18E-05 | 2.88E-09 |
|  | rs1260326 | 2 | 27508073 | C | T | -0.146 | 0.032 | 7.26E-06 | 0.068 | 0.017 | 8.24E-05 | 1.89E-08 |
|  | rs780093 | 2 | 27519736 | C | T | -0.157 | 0.032 | 1.29E-06 | 0.071 | 0.017 | 3.72E-05 | 1.93E-09 |
|  | rs1260333 | 2 | 27525757 | G | A | -0.138 | 0.032 | 1.43E-05 | 0.074 | 0.018 | 3.11E-05 | 1.26E-08 |
| CD-Stroke | rs76024230 | 7 | 5534865 | T | C | 0.168 | 0.035 | 1.80E-06 | 0.001 | 0.000 | 4.50E-04 | 2.12E-08 |
| UC-PD | rs147288664 | 1 | 152220451 | G | C | 0.258 | 0.077 | 7.73E-04 | -1.178 | 0.157 | 6.33E-14 | 1.18E-15 |
|  | rs12120358 | 1 | 161474579 | T | A | -0.109 | 0.019 | 1.18E-08 | 0.067 | 0.020 | 5.52E-04 | 2.74E-10 |
|  | rs6694403 | 1 | 161490421 | A | G | -0.114 | 0.019 | 7.56E-10 | 0.067 | 0.018 | 1.92E-04 | 6.91E-12 |
|  | rs4657039 | 1 | 161492938 | G | A | -0.109 | 0.019 | 4.76E-09 | 0.068 | 0.018 | 1.68E-04 | 2.38E-11 |
|  | rs7511868 | 1 | 161493541 | A | G | -0.111 | 0.019 | 2.31E-09 | 0.066 | 0.018 | 2.49E-04 | 2.25E-11 |
|  | rs7535475 | 1 | 161493797 | A | C | -0.112 | 0.019 | 1.52E-09 | 0.066 | 0.018 | 2.56E-04 | 1.72E-11 |
|  | rs7512140 | 1 | 161493811 | A | G | -0.115 | 0.019 | 7.90E-10 | 0.073 | 0.018 | 6.71E-05 | 1.52E-12 |
|  | rs6658353 | 1 | 161499264 | C | G | -0.162 | 0.019 | 1.99E-18 | 0.072 | 0.017 | 2.42E-05 | 9.90E-20 |
|  | rs7551957 | 1 | 161500252 | C | T | -0.165 | 0.019 | 1.54E-18 | 0.067 | 0.017 | 8.32E-05 | 3.06E-19 |
|  | rs12139150 | 1 | 161500715 | C | G | -0.165 | 0.019 | 1.53E-18 | 0.067 | 0.017 | 8.53E-05 | 3.03E-19 |
|  | rs6671847 | 1 | 161509020 | A | G | -0.164 | 0.019 | 2.05E-18 | 0.061 | 0.018 | 6.58E-04 | 4.18E-19 |
|  | rs4657041 | 1 | 161509069 | C | T | -0.162 | 0.019 | 2.12E-18 | 0.071 | 0.017 | 3.53E-05 | 2.02E-19 |
|  | rs1801274 | 1 | 161509955 | G | A | -0.163 | 0.018 | 1.37E-18 | 0.070 | 0.017 | 4.38E-05 | 2.08E-19 |
|  | rs71632960 | 1 | 161546570 | A | G | 0.223 | 0.041 | 4.71E-08 | -0.168 | 0.049 | 5.61E-04 | 7.97E-10 |
|  | rs3791304 | 2 | 134411105 | G | A | 0.075 | 0.019 | 8.11E-05 | 0.085 | 0.021 | 6.61E-05 | 3.71E-08 |
|  | rs499582 | 2 | 134433794 | G | A | -0.077 | 0.019 | 5.33E-05 | -0.090 | 0.021 | 2.48E-05 | 7.81E-09 |
|  | rs557408 | 2 | 134442145 | G | A | -0.079 | 0.019 | 3.64E-05 | -0.091 | 0.021 | 1.68E-05 | 3.59E-09 |
|  | rs598954 | 2 | 134442530 | C | T | -0.073 | 0.019 | 9.47E-05 | -0.089 | 0.020 | 1.41E-05 | 7.40E-09 |
|  | rs661899 | 2 | 134445591 | C | T | -0.080 | 0.019 | 2.19E-05 | -0.091 | 0.021 | 1.26E-05 | 1.54E-09 |
|  | rs629794 | 2 | 134448164 | G | A | -0.076 | 0.019 | 4.78E-05 | -0.084 | 0.021 | 4.77E-05 | 1.44E-08 |
|  | rs651970 | 2 | 134450506 | G | A | -0.076 | 0.019 | 5.21E-05 | -0.083 | 0.021 | 5.31E-05 | 1.80E-08 |
|  | rs626540 | 2 | 134450642 | G | A | -0.077 | 0.019 | 3.93E-05 | -0.084 | 0.021 | 5.02E-05 | 1.28E-08 |
|  | rs842354 | 2 | 134586364 | G | C | -0.093 | 0.019 | 6.81E-07 | -0.092 | 0.018 | 1.94E-07 | 4.70E-13 |
|  | rs842359 | 2 | 134587998 | C | G | -0.097 | 0.019 | 2.33E-07 | -0.092 | 0.018 | 1.69E-07 | 1.27E-13 |
|  | rs13410301 | 2 | 134595613 | T | C | 0.090 | 0.019 | 1.87E-06 | 0.082 | 0.018 | 3.07E-06 | 2.78E-11 |
|  | rs6707326 | 2 | 134600135 | A | G | 0.097 | 0.019 | 2.25E-07 | 0.096 | 0.018 | 4.83E-08 | 3.54E-14 |
|  | rs10170773 | 2 | 134604935 | C | T | 0.091 | 0.019 | 1.69E-06 | 0.086 | 0.018 | 9.58E-07 | 7.12E-12 |
|  | rs35215000 | 2 | 134610086 | A | C | 0.090 | 0.019 | 1.76E-06 | 0.086 | 0.018 | 8.79E-07 | 6.70E-12 |
|  | rs10171371 | 2 | 134614589 | T | C | 0.093 | 0.019 | 7.60E-07 | 0.085 | 0.018 | 1.18E-06 | 3.98E-12 |
|  | rs35564151 | 2 | 134615381 | A | G | 0.092 | 0.019 | 7.73E-07 | 0.093 | 0.018 | 3.02E-07 | 8.50E-13 |
|  | rs6728095 | 2 | 134618502 | A | T | 0.091 | 0.019 | 1.62E-06 | 0.086 | 0.018 | 9.17E-07 | 6.62E-12 |
|  | rs17789633 | 2 | 134628904 | C | A | 0.093 | 0.019 | 9.55E-07 | 0.085 | 0.018 | 1.09E-06 | 4.65E-12 |
|  | rs13399952 | 2 | 134636834 | G | A | 0.090 | 0.019 | 1.92E-06 | 0.087 | 0.018 | 7.04E-07 | 6.02E-12 |
|  | rs10928507 | 2 | 134639061 | G | A | 0.090 | 0.019 | 1.92E-06 | 0.087 | 0.018 | 6.77E-07 | 5.80E-12 |
|  | rs6705916 | 2 | 134639999 | T | C | 0.090 | 0.019 | 1.89E-06 | 0.086 | 0.017 | 7.87E-07 | 5.73E-12 |
|  | rs10193171 | 2 | 134645629 | G | A | 0.090 | 0.019 | 1.92E-06 | 0.086 | 0.017 | 9.21E-07 | 7.08E-12 |
|  | rs13424016 | 2 | 134648825 | G | A | 0.096 | 0.019 | 2.92E-07 | 0.095 | 0.018 | 5.83E-08 | 5.41E-14 |
|  | rs1568120 | 2 | 134649320 | G | A | 0.090 | 0.019 | 1.94E-06 | 0.085 | 0.017 | 1.04E-06 | 8.19E-12 |
|  | rs6724774 | 2 | 134649715 | T | C | 0.090 | 0.019 | 1.93E-06 | 0.085 | 0.018 | 1.17E-06 | 9.23E-12 |
|  | rs6724777 | 2 | 134649722 | T | C | 0.090 | 0.019 | 1.93E-06 | 0.086 | 0.018 | 1.10E-06 | 8.64E-12 |
|  | rs6724866 | 2 | 134649740 | A | C | 0.090 | 0.019 | 1.92E-06 | 0.085 | 0.018 | 1.43E-06 | 1.20E-11 |
|  | rs6752634 | 2 | 134649785 | A | G | 0.090 | 0.019 | 1.92E-06 | 0.086 | 0.018 | 1.10E-06 | 9.79E-12 |
|  | rs6742638 | 2 | 134649790 | G | T | 0.090 | 0.019 | 1.92E-06 | 0.086 | 0.018 | 1.10E-06 | 9.79E-12 |
|  | rs6739706 | 2 | 134649839 | C | A | 0.090 | 0.019 | 1.92E-06 | 0.085 | 0.018 | 1.18E-06 | 1.02E-11 |
|  | rs12691869 | 2 | 134650071 | C | T | 0.090 | 0.019 | 2.00E-06 | 0.086 | 0.018 | 7.36E-07 | 6.66E-12 |
|  | rs10210052 | 2 | 134659780 | T | C | 0.089 | 0.019 | 2.44E-06 | 0.087 | 0.018 | 5.69E-07 | 5.84E-12 |
|  | rs11688414 | 2 | 134662667 | A | G | 0.096 | 0.019 | 3.42E-07 | 0.097 | 0.018 | 2.88E-08 | 2.67E-14 |
|  | rs998451 | 2 | 134671718 | A | G | 0.101 | 0.019 | 7.42E-08 | 0.081 | 0.018 | 9.63E-06 | 6.11E-12 |
|  | rs6745983 | 2 | 134673139 | A | G | 0.090 | 0.019 | 2.31E-06 | 0.089 | 0.018 | 4.04E-07 | 3.60E-12 |
|  | rs1123184 | 2 | 134673487 | G | C | 0.090 | 0.019 | 2.31E-06 | 0.088 | 0.018 | 4.62E-07 | 4.12E-12 |
|  | rs11898465 | 2 | 134673879 | T | G | 0.090 | 0.019 | 2.33E-06 | 0.089 | 0.018 | 4.38E-07 | 3.88E-12 |
|  | rs4954160 | 2 | 134674122 | C | A | 0.090 | 0.019 | 2.34E-06 | 0.089 | 0.018 | 4.10E-07 | 3.64E-12 |
|  | rs4954161 | 2 | 134674413 | G | A | 0.089 | 0.019 | 2.38E-06 | 0.088 | 0.018 | 4.90E-07 | 4.53E-12 |
|  | rs35570087 | 2 | 134674610 | A | G | 0.090 | 0.019 | 2.33E-06 | 0.088 | 0.018 | 5.05E-07 | 4.58E-12 |
|  | rs883964 | 2 | 134678630 | C | G | 0.096 | 0.020 | 1.29E-06 | 0.060 | 0.018 | 8.81E-04 | 2.23E-08 |
|  | rs1104802 | 2 | 134678796 | C | A | 0.096 | 0.020 | 1.29E-06 | 0.059 | 0.018 | 9.71E-04 | 2.54E-08 |
|  | rs1104801 | 2 | 134679031 | A | G | 0.093 | 0.020 | 2.78E-06 | 0.060 | 0.018 | 9.23E-04 | 4.50E-08 |
|  | rs10171677 | 2 | 134688820 | G | A | 0.090 | 0.019 | 1.60E-06 | 0.094 | 0.018 | 2.18E-07 | 1.23E-12 |
|  | rs10928512 | 2 | 134693732 | T | G | 0.081 | 0.019 | 2.14E-05 | 0.107 | 0.018 | 1.13E-09 | 9.26E-14 |
|  | rs10928513 | 2 | 134699189 | T | C | -0.098 | 0.019 | 1.45E-07 | -0.085 | 0.018 | 3.26E-06 | 2.81E-12 |
|  | rs6723108 | 2 | 134722410 | T | G | -0.099 | 0.019 | 1.24E-07 | -0.091 | 0.018 | 6.54E-07 | 3.67E-13 |
|  | rs6756200 | 2 | 134723708 | C | T | -0.092 | 0.019 | 8.22E-07 | -0.098 | 0.018 | 6.39E-08 | 1.53E-13 |
|  | rs1942055 | 2 | 134741354 | C | G | -0.096 | 0.019 | 2.88E-07 | -0.092 | 0.019 | 7.17E-07 | 9.30E-13 |
|  | rs1942041 | 2 | 134757922 | C | T | -0.093 | 0.019 | 1.34E-06 | -0.119 | 0.018 | 1.49E-11 | 4.29E-17 |
|  | rs6741007 | 2 | 134779549 | G | T | -0.086 | 0.020 | 1.36E-05 | -0.123 | 0.018 | 2.09E-12 | 9.36E-17 |
|  | rs6751833 | 2 | 134780328 | T | G | -0.083 | 0.020 | 2.68E-05 | -0.120 | 0.018 | 9.38E-12 | 1.17E-15 |
|  | rs6430538 | 2 | 134782397 | T | C | -0.085 | 0.020 | 1.58E-05 | -0.123 | 0.018 | 2.57E-12 | 1.55E-16 |
|  | rs7599054 | 2 | 134782976 | G | A | -0.086 | 0.020 | 1.47E-05 | -0.122 | 0.018 | 2.80E-12 | 1.47E-16 |
|  | rs6753334 | 2 | 134818984 | G | A | -0.085 | 0.020 | 1.70E-05 | -0.120 | 0.018 | 1.05E-11 | 6.83E-16 |
|  | rs4953936 | 2 | 134823942 | T | C | -0.085 | 0.020 | 1.79E-05 | -0.119 | 0.018 | 1.38E-11 | 8.48E-16 |
|  | rs6758044 | 2 | 134834675 | C | T | -0.085 | 0.020 | 1.97E-05 | -0.108 | 0.019 | 1.78E-08 | 1.22E-12 |
|  | rs10496731 | 2 | 134840058 | G | T | -0.070 | 0.019 | 2.49E-04 | -0.081 | 0.017 | 2.93E-06 | 4.09E-09 |
|  | rs1893396 | 2 | 134841439 | T | G | -0.076 | 0.019 | 5.55E-05 | -0.080 | 0.017 | 4.16E-06 | 1.12E-09 |
|  | rs3739029 | 2 | 134841837 | C | T | -0.070 | 0.019 | 2.05E-04 | -0.082 | 0.017 | 2.58E-06 | 2.79E-09 |
|  | rs1530556 | 2 | 134842164 | T | C | -0.071 | 0.019 | 1.97E-04 | -0.080 | 0.017 | 3.96E-06 | 4.11E-09 |
|  | rs1530555 | 2 | 134842169 | T | C | -0.070 | 0.019 | 2.05E-04 | -0.082 | 0.017 | 2.59E-06 | 2.79E-09 |
|  | rs6729702 | 2 | 134844586 | A | G | -0.071 | 0.019 | 2.17E-04 | -0.081 | 0.017 | 3.07E-06 | 3.63E-09 |
|  | rs6430543 | 2 | 134844839 | A | T | -0.071 | 0.019 | 2.17E-04 | -0.082 | 0.017 | 2.53E-06 | 2.97E-09 |
|  | rs6430545 | 2 | 134845031 | A | T | -0.075 | 0.019 | 6.22E-05 | -0.082 | 0.017 | 2.72E-06 | 8.05E-10 |
|  | rs1942051 | 2 | 134846120 | G | A | -0.075 | 0.019 | 6.22E-05 | -0.081 | 0.018 | 3.96E-06 | 1.36E-09 |
|  | rs10928519 | 2 | 134846389 | T | C | -0.075 | 0.019 | 6.21E-05 | -0.080 | 0.018 | 4.06E-06 | 1.39E-09 |
|  | rs10928520 | 2 | 134846613 | C | T | -0.071 | 0.019 | 2.18E-04 | -0.082 | 0.017 | 2.51E-06 | 2.98E-09 |
|  | rs1530557 | 2 | 134849309 | C | T | -0.070 | 0.019 | 2.28E-04 | -0.081 | 0.017 | 3.02E-06 | 3.72E-09 |
|  | rs4954189 | 2 | 134849763 | A | G | -0.075 | 0.019 | 6.49E-05 | -0.081 | 0.017 | 3.31E-06 | 1.01E-09 |
|  | rs56383182 | 2 | 134850641 | G | A | -0.070 | 0.019 | 2.28E-04 | -0.081 | 0.017 | 3.04E-06 | 3.71E-09 |
|  | rs6430546 | 2 | 134851231 | T | A | -0.070 | 0.019 | 2.28E-04 | -0.081 | 0.017 | 3.42E-06 | 4.29E-09 |
|  | rs6730306 | 2 | 134851597 | T | C | -0.070 | 0.019 | 2.28E-04 | -0.081 | 0.017 | 3.46E-06 | 4.29E-09 |
|  | rs6758113 | 2 | 134851677 | A | G | -0.071 | 0.019 | 1.80E-04 | -0.078 | 0.017 | 7.97E-06 | 7.83E-09 |
|  | rs60793693 | 2 | 134852125 | T | G | -0.070 | 0.019 | 2.28E-04 | -0.081 | 0.017 | 3.47E-06 | 4.29E-09 |
|  | rs10432371 | 2 | 134852176 | A | G | -0.075 | 0.019 | 6.46E-05 | -0.081 | 0.017 | 3.20E-06 | 9.71E-10 |
|  | rs2053726 | 2 | 134852584 | G | A | -0.075 | 0.019 | 6.50E-05 | -0.080 | 0.018 | 4.67E-06 | 1.69E-09 |
|  | rs6430547 | 2 | 134854852 | T | C | -0.071 | 0.019 | 1.80E-04 | -0.077 | 0.018 | 1.11E-05 | 1.17E-08 |
|  | rs9653176 | 2 | 134857306 | T | C | -0.070 | 0.019 | 2.28E-04 | -0.087 | 0.018 | 1.60E-06 | 2.04E-09 |
|  | rs6430549 | 2 | 134858776 | G | A | -0.075 | 0.019 | 7.02E-05 | -0.081 | 0.018 | 3.09E-06 | 1.18E-09 |
|  | rs6430550 | 2 | 134858815 | T | C | -0.074 | 0.019 | 7.04E-05 | -0.080 | 0.018 | 4.37E-06 | 1.68E-09 |
|  | rs1446523 | 2 | 134859051 | A | G | -0.075 | 0.019 | 6.69E-05 | -0.082 | 0.018 | 3.05E-06 | 1.09E-09 |
|  | rs6430551 | 2 | 134863986 | C | T | -0.071 | 0.019 | 1.59E-04 | -0.082 | 0.018 | 2.69E-06 | 2.42E-09 |
|  | rs6430552 | 2 | 134865238 | T | C | -0.067 | 0.019 | 4.78E-04 | -0.080 | 0.017 | 4.14E-06 | 1.14E-08 |
|  | rs3819121 | 2 | 134865290 | C | T | -0.069 | 0.019 | 2.98E-04 | -0.077 | 0.017 | 1.15E-05 | 1.91E-08 |
|  | rs7593370 | 2 | 134865947 | T | C | -0.072 | 0.019 | 1.14E-04 | -0.082 | 0.017 | 2.99E-06 | 1.67E-09 |
|  | rs3814358 | 2 | 134866438 | C | A | -0.072 | 0.019 | 1.14E-04 | -0.089 | 0.018 | 1.05E-06 | 5.57E-10 |
|  | rs7580655 | 2 | 134869605 | T | G | -0.068 | 0.019 | 3.56E-04 | -0.080 | 0.017 | 3.97E-06 | 7.81E-09 |
|  | rs6711390 | 2 | 134871869 | T | C | -0.067 | 0.019 | 4.14E-04 | -0.080 | 0.017 | 3.87E-06 | 9.23E-09 |
|  | rs12469941 | 2 | 134872357 | T | C | -0.072 | 0.019 | 1.13E-04 | -0.081 | 0.018 | 3.73E-06 | 2.39E-09 |
|  | rs1446524 | 2 | 134873761 | G | A | -0.072 | 0.019 | 1.13E-04 | -0.081 | 0.018 | 3.66E-06 | 2.32E-09 |
|  | rs6430553 | 2 | 134873830 | T | C | -0.067 | 0.019 | 4.33E-04 | -0.080 | 0.017 | 3.87E-06 | 9.69E-09 |
|  | rs4954192 | 2 | 134875411 | T | C | -0.067 | 0.019 | 4.52E-04 | -0.082 | 0.017 | 2.64E-06 | 6.85E-09 |
|  | rs6738113 | 2 | 134877011 | A | G | -0.067 | 0.019 | 4.48E-04 | -0.080 | 0.017 | 4.19E-06 | 1.09E-08 |
|  | rs2166480 | 2 | 134879768 | A | G | -0.072 | 0.019 | 1.26E-04 | -0.081 | 0.017 | 3.63E-06 | 2.27E-09 |
|  | rs1446525 | 2 | 134880277 | A | G | -0.072 | 0.019 | 1.32E-04 | -0.081 | 0.017 | 3.70E-06 | 2.46E-09 |
|  | rs12473839 | 2 | 134880474 | T | C | -0.067 | 0.019 | 4.49E-04 | -0.080 | 0.017 | 4.85E-06 | 1.26E-08 |
|  | rs6430554 | 2 | 134880891 | T | C | -0.072 | 0.019 | 1.23E-04 | -0.081 | 0.017 | 3.72E-06 | 2.29E-09 |
|  | rs1348790 | 2 | 134882405 | G | C | -0.072 | 0.019 | 1.23E-04 | -0.080 | 0.017 | 4.57E-06 | 2.80E-09 |
|  | rs4954193 | 2 | 134884610 | G | A | -0.073 | 0.019 | 1.09E-04 | -0.080 | 0.017 | 4.25E-06 | 2.33E-09 |
|  | rs111674445 | 2 | 134885149 | A | T | -0.067 | 0.019 | 4.05E-04 | -0.080 | 0.017 | 4.50E-06 | 1.04E-08 |
|  | rs62168869 | 2 | 134889209 | A | G | -0.067 | 0.019 | 4.15E-04 | -0.080 | 0.017 | 4.37E-06 | 1.04E-08 |
|  | rs6430555 | 2 | 134890089 | C | A | -0.073 | 0.019 | 1.05E-04 | -0.088 | 0.018 | 1.23E-06 | 6.25E-10 |
|  | rs74265413 | 2 | 134892083 | C | G | -0.069 | 0.019 | 3.03E-04 | -0.076 | 0.017 | 1.26E-05 | 2.17E-08 |
|  | rs6714498 | 2 | 134893196 | C | T | -0.067 | 0.019 | 4.02E-04 | -0.077 | 0.017 | 1.03E-05 | 2.36E-08 |
|  | rs7589297 | 2 | 134894829 | A | C | -0.073 | 0.019 | 1.08E-04 | -0.081 | 0.017 | 3.23E-06 | 1.72E-09 |
|  | rs7593049 | 2 | 134895785 | T | C | -0.068 | 0.019 | 3.53E-04 | -0.076 | 0.017 | 1.33E-05 | 2.70E-08 |
|  | rs1374292 | 2 | 134896598 | A | G | -0.072 | 0.019 | 1.14E-04 | -0.080 | 0.018 | 4.95E-06 | 3.21E-09 |
|  | rs1374291 | 2 | 134896768 | C | T | -0.072 | 0.019 | 1.14E-04 | -0.081 | 0.017 | 3.62E-06 | 2.04E-09 |
|  | rs10176573 | 2 | 134899901 | A | C | -0.072 | 0.019 | 1.14E-04 | -0.082 | 0.017 | 2.94E-06 | 1.66E-09 |
|  | rs1348792 | 2 | 134903288 | G | A | -0.069 | 0.019 | 3.26E-04 | -0.076 | 0.017 | 1.22E-05 | 2.23E-08 |
|  | rs62168871 | 2 | 134904108 | G | A | -0.072 | 0.019 | 1.13E-04 | -0.081 | 0.017 | 3.23E-06 | 1.81E-09 |
|  | rs62168872 | 2 | 134904177 | T | C | -0.072 | 0.019 | 1.11E-04 | -0.081 | 0.017 | 3.23E-06 | 1.77E-09 |
|  | rs4954196 | 2 | 134904446 | G | A | -0.072 | 0.019 | 1.13E-04 | -0.081 | 0.017 | 3.31E-06 | 1.86E-09 |
|  | rs4954197 | 2 | 134904674 | A | G | -0.072 | 0.019 | 1.11E-04 | -0.081 | 0.017 | 3.24E-06 | 1.77E-09 |
|  | rs12469411 | 2 | 134906986 | T | C | -0.066 | 0.019 | 4.40E-04 | -0.080 | 0.017 | 4.63E-06 | 1.14E-08 |
|  | rs10928523 | 2 | 134907891 | T | C | -0.072 | 0.019 | 1.13E-04 | -0.080 | 0.017 | 4.60E-06 | 2.63E-09 |
|  | rs6733130 | 2 | 134908983 | C | A | -0.072 | 0.019 | 1.13E-04 | -0.080 | 0.017 | 4.56E-06 | 2.64E-09 |
|  | rs766270 | 2 | 134909356 | C | G | -0.073 | 0.019 | 1.04E-04 | -0.081 | 0.017 | 3.27E-06 | 1.70E-09 |
|  | rs1374288 | 2 | 134909483 | T | C | -0.073 | 0.019 | 1.05E-04 | -0.087 | 0.018 | 1.66E-06 | 8.52E-10 |
|  | rs766271 | 2 | 134909561 | T | C | -0.073 | 0.019 | 1.05E-04 | -0.080 | 0.017 | 4.61E-06 | 2.50E-09 |
|  | rs1374287 | 2 | 134909631 | T | C | -0.073 | 0.019 | 1.04E-04 | -0.080 | 0.017 | 4.58E-06 | 2.41E-09 |
|  | rs2322251 | 2 | 134913732 | T | C | -0.072 | 0.019 | 1.11E-04 | -0.081 | 0.017 | 3.16E-06 | 1.78E-09 |
|  | rs12463645 | 2 | 134916800 | C | T | -0.069 | 0.019 | 3.29E-04 | -0.077 | 0.017 | 1.15E-05 | 2.12E-08 |
|  | rs6738563 | 2 | 134931618 | T | G | -0.083 | 0.019 | 8.94E-06 | -0.074 | 0.020 | 2.00E-04 | 1.75E-08 |
|  | rs4953938 | 2 | 134937278 | A | G | -0.075 | 0.019 | 6.94E-05 | -0.082 | 0.017 | 2.44E-06 | 8.25E-10 |
|  | rs10187990 | 2 | 134939987 | G | A | -0.075 | 0.019 | 6.57E-05 | -0.082 | 0.017 | 2.40E-06 | 7.56E-10 |
|  | rs1979033 | 2 | 134947988 | A | G | -0.083 | 0.019 | 8.88E-06 | -0.081 | 0.018 | 7.86E-06 | 3.71E-10 |
|  | rs2034261 | 2 | 134949188 | T | G | -0.075 | 0.019 | 6.92E-05 | -0.082 | 0.017 | 2.29E-06 | 7.52E-10 |
|  | rs10166142 | 2 | 134952752 | G | A | -0.074 | 0.019 | 7.26E-05 | -0.091 | 0.018 | 6.00E-07 | 2.01E-10 |
|  | rs3814355 | 2 | 134953946 | T | C | -0.075 | 0.019 | 6.86E-05 | -0.082 | 0.017 | 2.45E-06 | 8.16E-10 |
|  | rs3814354 | 2 | 134954087 | A | G | -0.075 | 0.019 | 6.95E-05 | -0.084 | 0.017 | 1.58E-06 | 5.11E-10 |
|  | rs3769023 | 2 | 134956133 | A | G | -0.074 | 0.019 | 7.49E-05 | -0.084 | 0.017 | 1.57E-06 | 5.53E-10 |
|  | rs1947112 | 2 | 134958387 | G | A | -0.075 | 0.019 | 6.65E-05 | -0.084 | 0.017 | 1.62E-06 | 5.03E-10 |
|  | rs62168897 | 2 | 134960427 | T | C | -0.075 | 0.019 | 6.86E-05 | -0.084 | 0.017 | 1.64E-06 | 5.19E-10 |
|  | rs6708336 | 2 | 134961562 | A | T | -0.075 | 0.019 | 6.90E-05 | -0.084 | 0.017 | 1.67E-06 | 5.39E-10 |
|  | rs6708336 | 2 | 134961562 | A | T | -0.075 | 0.019 | 6.90E-05 | -0.084 | 0.017 | 1.67E-06 | 5.39E-10 |
|  | rs56271357 | 2 | 134963145 | T | C | -0.075 | 0.019 | 6.89E-05 | -0.084 | 0.017 | 1.65E-06 | 5.22E-10 |
|  | rs1592 | 2 | 134964573 | C | A | -0.075 | 0.019 | 6.33E-05 | -0.085 | 0.018 | 1.11E-06 | 3.61E-10 |
|  | rs28413725 | 3 | 46489195 | T | C | 0.100 | 0.019 | 7.85E-08 | 0.059 | 0.018 | 9.63E-04 | 2.77E-09 |
|  | rs9845968 | 3 | 46489654 | G | A | 0.068 | 0.019 | 4.06E-04 | 0.084 | 0.018 | 1.42E-06 | 3.51E-09 |
|  | rs12496864 | 3 | 46493581 | T | C | 0.100 | 0.019 | 7.57E-08 | 0.062 | 0.018 | 5.23E-04 | 1.15E-09 |
|  | rs867620 | 3 | 46496359 | T | C | 0.069 | 0.019 | 3.49E-04 | 0.084 | 0.018 | 1.56E-06 | 3.23E-09 |
|  | rs883739 | 3 | 46496528 | T | G | 0.101 | 0.019 | 6.14E-08 | 0.061 | 0.018 | 6.25E-04 | 1.26E-09 |
|  | rs2037391 | 3 | 46498033 | A | G | 0.101 | 0.019 | 6.35E-08 | 0.062 | 0.018 | 5.46E-04 | 1.09E-09 |
|  | rs10865942 | 3 | 46499442 | C | G | 0.078 | 0.019 | 3.65E-05 | 0.077 | 0.017 | 9.98E-06 | 1.91E-09 |
|  | rs7430431 | 3 | 46499657 | C | T | 0.068 | 0.019 | 3.92E-04 | 0.083 | 0.018 | 1.94E-06 | 4.62E-09 |
|  | rs12490169 | 3 | 46505910 | T | C | 0.100 | 0.019 | 8.53E-08 | 0.060 | 0.018 | 8.73E-04 | 2.57E-09 |
|  | rs60986461 | 3 | 46506556 | T | C | 0.100 | 0.019 | 8.56E-08 | 0.060 | 0.018 | 8.21E-04 | 2.37E-09 |
|  | rs115891767 | 4 | 89592368 | G | T | -0.220 | 0.067 | 9.66E-04 | 0.227 | 0.044 | 2.80E-07 | 1.80E-09 |
|  | rs182585138 | 4 | 89602489 | C | T | -0.219 | 0.067 | 9.71E-04 | 0.228 | 0.044 | 2.25E-07 | 1.39E-09 |
|  | rs144189779 | 7 | 4739652 | C | G | 0.276 | 0.047 | 3.19E-09 | -0.595 | 0.175 | 6.84E-04 | 1.31E-10 |
|  | rs11175620 | 12 | 40217062 | C | G | 0.136 | 0.037 | 2.19E-04 | 0.121 | 0.025 | 1.46E-06 | 1.74E-09 |
|  | rs11175645 | 12 | 40228090 | G | A | 0.136 | 0.037 | 2.36E-04 | 0.120 | 0.025 | 2.28E-06 | 2.88E-09 |
|  | rs11175658 | 12 | 40230508 | G | A | 0.136 | 0.037 | 2.31E-04 | 0.120 | 0.025 | 2.31E-06 | 2.81E-09 |
|  | rs74324737 | 12 | 40231279 | A | G | 0.136 | 0.037 | 2.27E-04 | 0.120 | 0.025 | 2.40E-06 | 2.86E-09 |
|  | rs11175666 | 12 | 40234631 | T | C | 0.137 | 0.037 | 2.21E-04 | 0.120 | 0.025 | 2.24E-06 | 2.77E-09 |
|  | rs6011026 | 20 | 63677133 | G | A | 0.092 | 0.019 | 1.05E-06 | -0.075 | 0.022 | 5.47E-04 | 9.64E-09 |
| UC-MS | rs13003464 | 2 | 60959694 | G | A | 0.102 | 0.019 | 8.70E-08 | 0.000 | 0.000 | 2.70E-04 | 2.28E-09 |
|  | rs67927699 | 2 | 60960280 | C | G | 0.104 | 0.019 | 5.41E-08 | 0.000 | 0.000 | 1.80E-04 | 9.96E-10 |
|  | rs10182512 | 2 | 60962334 | A | G | 0.102 | 0.019 | 1.31E-07 | 0.001 | 0.000 | 1.40E-04 | 1.39E-09 |
|  | rs10195112 | 2 | 60962605 | A | T | 0.102 | 0.019 | 1.07E-07 | 0.000 | 0.000 | 1.60E-04 | 1.40E-09 |
|  | rs55776317 | 2 | 60964860 | C | G | 0.102 | 0.019 | 1.13E-07 | 0.001 | 0.000 | 8.90E-05 | 7.37E-10 |
|  | rs10191548 | 2 | 60970260 | A | G | 0.101 | 0.019 | 1.48E-07 | 0.000 | 0.000 | 1.90E-04 | 2.21E-09 |
|  | rs112401990 | 2 | 60972192 | A | G | 0.104 | 0.019 | 8.09E-08 | 0.001 | 0.000 | 1.10E-04 | 7.00E-10 |
|  | rs1962449 | 2 | 60975414 | G | C | 0.103 | 0.019 | 9.84E-08 | 0.001 | 0.000 | 6.50E-05 | 4.60E-10 |
|  | rs7608697 | 2 | 60977506 | C | A | 0.103 | 0.019 | 6.27E-08 | 0.000 | 0.000 | 1.60E-04 | 9.51E-10 |
|  | rs7596362 | 2 | 60977553 | C | T | 0.103 | 0.019 | 6.27E-08 | 0.000 | 0.000 | 2.00E-04 | 1.26E-09 |
|  | rs7608910 | 2 | 60977721 | G | A | 0.105 | 0.019 | 3.70E-08 | 0.000 | 0.000 | 1.40E-04 | 5.12E-10 |
|  | rs4560096 | 2 | 60977782 | G | T | 0.103 | 0.019 | 6.27E-08 | 0.000 | 0.000 | 1.50E-04 | 8.62E-10 |
|  | rs7562347 | 2 | 60982740 | G | T | 0.093 | 0.019 | 4.95E-07 | 0.001 | 0.000 | 3.80E-05 | 9.16E-10 |
|  | rs747854 | 14 | 68767119 | A | G | -0.065 | 0.019 | 8.23E-04 | -0.001 | 0.000 | 2.40E-07 | 7.20E-09 |
|  | rs3869550 | 17 | 42340869 | C | T | -0.095 | 0.019 | 6.74E-07 | 0.000 | 0.000 | 1.60E-04 | 1.44E-09 |
|  | rs8069645 | 17 | 42342884 | G | A | -0.098 | 0.021 | 2.72E-06 | 0.001 | 0.000 | 2.20E-04 | 7.33E-09 |
|  | rs8072391 | 17 | 42343372 | A | G | -0.096 | 0.019 | 5.35E-07 | 0.000 | 0.000 | 1.80E-04 | 1.41E-09 |
|  | rs7217655 | 17 | 42344006 | T | C | -0.096 | 0.019 | 5.42E-07 | 0.000 | 0.000 | 1.70E-04 | 1.32E-09 |
|  | rs17886724 | 17 | 42344145 | G | A | -0.096 | 0.019 | 5.42E-07 | 0.000 | 0.000 | 1.70E-04 | 1.33E-09 |
|  | rs12950549 | 17 | 42344576 | G | A | -0.096 | 0.019 | 5.41E-07 | 0.000 | 0.000 | 1.60E-04 | 1.24E-09 |
|  | rs12601611 | 17 | 42345810 | T | C | -0.099 | 0.021 | 2.39E-06 | 0.001 | 0.000 | 1.90E-04 | 5.51E-09 |
|  | rs3736162 | 17 | 42345821 | C | G | -0.099 | 0.021 | 2.39E-06 | 0.001 | 0.000 | 2.00E-04 | 5.78E-09 |
|  | rs3816769 | 17 | 42346255 | C | T | -0.096 | 0.019 | 6.35E-07 | 0.000 | 0.000 | 1.70E-04 | 1.50E-09 |
|  | rs17885741 | 17 | 42346926 | C | T | -0.095 | 0.019 | 6.71E-07 | 0.000 | 0.000 | 1.70E-04 | 1.56E-09 |
|  | rs8081037 | 17 | 42347140 | T | C | -0.096 | 0.019 | 5.37E-07 | 0.000 | 0.000 | 1.80E-04 | 1.46E-09 |
|  | rs6503696 | 17 | 42347786 | T | C | -0.099 | 0.021 | 2.47E-06 | 0.001 | 0.000 | 2.20E-04 | 6.84E-09 |
|  | rs2306581 | 17 | 42348247 | A | C | -0.096 | 0.019 | 5.40E-07 | 0.000 | 0.000 | 2.00E-04 | 1.60E-09 |
|  | rs6503697 | 17 | 42349561 | T | A | -0.098 | 0.021 | 2.68E-06 | 0.001 | 0.000 | 2.30E-04 | 7.45E-09 |
|  | rs8073517 | 17 | 42351306 | C | T | -0.096 | 0.019 | 6.50E-07 | 0.000 | 0.000 | 2.00E-04 | 1.94E-09 |
|  | rs62075772 | 17 | 42352232 | G | A | -0.096 | 0.019 | 6.33E-07 | 0.000 | 0.000 | 1.80E-04 | 1.58E-09 |
|  | rs8076051 | 17 | 42353116 | G | A | -0.096 | 0.019 | 5.13E-07 | 0.000 | 0.000 | 1.80E-04 | 1.33E-09 |
|  | rs8075676 | 17 | 42353184 | T | C | -0.098 | 0.021 | 2.58E-06 | 0.001 | 0.000 | 2.50E-04 | 8.06E-09 |
|  | rs4103200 | 17 | 42355047 | C | G | -0.099 | 0.021 | 2.48E-06 | 0.001 | 0.000 | 2.30E-04 | 7.10E-09 |
|  | rs9891119 | 17 | 42355962 | C | A | -0.097 | 0.019 | 5.00E-07 | 0.000 | 0.000 | 1.80E-04 | 1.34E-09 |
|  | rs9913597 | 17 | 42358298 | C | T | -0.096 | 0.019 | 6.27E-07 | 0.000 | 0.000 | 2.30E-04 | 2.15E-09 |
|  | rs3785898 | 17 | 42363102 | A | C | -0.098 | 0.021 | 2.51E-06 | 0.001 | 0.000 | 2.50E-04 | 7.90E-09 |
|  | rs9895473 | 17 | 42363704 | A | G | -0.098 | 0.021 | 3.03E-06 | 0.001 | 0.000 | 2.50E-04 | 9.45E-09 |
|  | rs35314169 | 17 | 42363808 | T | C | -0.096 | 0.021 | 4.40E-06 | 0.001 | 0.000 | 2.50E-04 | 1.27E-08 |
|  | rs957970 | 17 | 42367872 | G | A | -0.096 | 0.019 | 6.27E-07 | 0.000 | 0.000 | 1.90E-04 | 1.77E-09 |
|  | rs957971 | 17 | 42367907 | G | C | 0.096 | 0.019 | 6.27E-07 | 0.000 | 0.000 | 2.50E-04 | 2.37E-09 |
|  | rs1905340 | 17 | 42368372 | A | C | -0.098 | 0.021 | 2.51E-06 | 0.001 | 0.000 | 2.70E-04 | 8.84E-09 |
|  | rs35840966 | 17 | 42369186 | T | C | -0.097 | 0.019 | 5.04E-07 | 0.000 | 0.000 | 1.90E-04 | 1.40E-09 |
|  | rs1026916 | 17 | 42377817 | G | A | 0.096 | 0.019 | 5.48E-07 | 0.000 | 0.000 | 2.00E-04 | 1.67E-09 |
|  | rs4796791 | 17 | 42378745 | C | T | 0.096 | 0.019 | 5.53E-07 | 0.000 | 0.000 | 1.80E-04 | 1.47E-09 |
|  | rs8071537 | 17 | 42378877 | T | G | -0.096 | 0.019 | 5.35E-07 | 0.000 | 0.000 | 1.90E-04 | 1.52E-09 |
|  | rs7219739 | 17 | 42379743 | T | G | -0.096 | 0.019 | 5.36E-07 | 0.000 | 0.000 | 1.90E-04 | 1.47E-09 |
|  | rs8068748 | 17 | 42380683 | C | T | -0.095 | 0.019 | 8.18E-07 | 0.000 | 0.000 | 1.80E-04 | 2.07E-09 |
|  | rs7211777 | 17 | 42382057 | A | G | 0.095 | 0.019 | 8.21E-07 | 0.000 | 0.000 | 2.30E-04 | 2.83E-09 |
|  | rs12942611 | 17 | 42383166 | A | C | -0.096 | 0.019 | 6.63E-07 | 0.000 | 0.000 | 2.90E-04 | 3.02E-09 |
|  | rs8070763 | 17 | 42384378 | C | T | -0.095 | 0.019 | 6.66E-07 | 0.000 | 0.000 | 2.90E-04 | 3.08E-09 |
|  | rs13342031 | 17 | 42384853 | G | C | -0.097 | 0.021 | 3.71E-06 | 0.000 | 0.000 | 4.40E-04 | 2.22E-08 |
|  | rs3736164 | 17 | 42387807 | G | T | 0.097 | 0.021 | 3.31E-06 | 0.000 | 0.000 | 4.40E-04 | 2.03E-08 |
|  | rs4796647 | 17 | 42391974 | A | G | 0.090 | 0.019 | 2.59E-06 | 0.000 | 0.000 | 4.10E-04 | 1.48E-08 |
| UC-Stroke | rs76024230 | 7 | 5534865 | T | C | 0.120 | 0.021 | 9.17E-09 | 0.001 | 0.000 | 4.50E-04 | 7.18E-10 |
| IBS-PD | rs4613561 | 4 | 15736267 | C | T | 0.054 | 0.016 | 8.90E-04 | -0.131 | 0.020 | 1.76E-11 | 9.64E-13 |
|  | rs4263397 | 4 | 15737767 | G | T | 0.056 | 0.016 | 6.88E-04 | -0.127 | 0.020 | 9.11E-11 | 3.15E-12 |
|  | rs2286879 | 4 | 15739257 | G | A | 0.060 | 0.017 | 4.15E-04 | -0.104 | 0.020 | 1.16E-07 | 1.22E-09 |
|  | rs10023845 | 4 | 15739628 | G | A | 0.063 | 0.017 | 2.65E-04 | -0.101 | 0.020 | 4.59E-07 | 3.30E-09 |
|  | rs10222984 | 4 | 15740080 | G | A | 0.063 | 0.017 | 2.54E-04 | -0.102 | 0.020 | 3.65E-07 | 2.33E-09 |
|  | rs3893376 | 4 | 15740451 | C | T | 0.063 | 0.017 | 2.61E-04 | -0.102 | 0.020 | 3.58E-07 | 2.33E-09 |
|  | rs12926804 | 16 | 71961033 | G | A | -0.087 | 0.025 | 5.76E-04 | -0.127 | 0.026 | 1.05E-06 | 1.60E-08 |
|  | rs34679758 | 16 | 71961528 | G | A | -0.087 | 0.025 | 5.80E-04 | -0.128 | 0.026 | 8.72E-07 | 1.34E-08 |

CD: Crohn's disease; UC: ulcerative colitis; IBS: irritable bowel syndrome; MS: multiple sclerosis; PD: Parkinson's disease; Chr: chromosome; SE: standard error.

**Supplementary Table 6. Novel pleiotropic loci between intestinal and neurological diseases in the non-MHC region (*p*_CPASSOC_<5×10^-8^, 5×10^-8^<*p*_single-trait_<1×10^-3^).**

| **Traits** | **SNP** | **Chr: Position** | **A1/A2** | **Trait 1** | | **Trait 2** | | ***p*_CPASSOC_** | **Gene** |
| --- | --- | --- | --- | --- | --- | --- | --- | --- | --- |
|  |  |  |  | **Beta** | ***p*** | **Beta** | ***p*** |  |  |
| CD-PD | rs780093 | Chr2:27519736 | C/T | -1.57E-01 | 1.29E-06 | 7.14E-02 | 3.72E-05 | 1.93E-09 | *GCKR* |
| CD-Stroke | rs76024230 | Chr7:5534865 | T/C | 1.68E-01 | 1.80E-06 | 1.07E-03 | 4.50E-04 | 2.12E-08 | *ACTB* |
| UC-PD | rs13424016 | Chr2:134648825 | G/A | 9.61E-02 | 2.92E-07 | 9.48E-02 | 5.83E-08 | 5.41E-14 | *TMEM163* |
|  | rs2037391 | Chr3:46498033 | A/G | 1.01E-01 | 6.35E-08 | 6.17E-02 | 5.46E-04 | 1.09E-09 | *RTP3* |
|  | rs182585138 | Chr4:89602489 | C/T | -2.19E-01 | 9.71E-04 | 2.28E-01 | 2.25E-07 | 1.39E-09 | *LOC105377329* |
|  | rs11175620 | Chr12:40217062 | C/G | 1.36E-01 | 2.19E-04 | 1.21E-01 | 1.46E-06 | 1.74E-09 | *LRRK2* |
|  | rs6011026 | Chr20:63677133 | G/A | 9.22E-02 | 1.05E-06 | -7.52E-02 | 5.47E-04 | 9.64E-09 | *RTEL1* |
| UC-MS | rs1962449 | Chr2:60975414 | G/C | 1.03E-01 | 9.84E-08 | 5.23E-04 | 6.50E-05 | 4.60E-10 | *PUS10* |
|  | rs747854 | Chr14:68767119 | A/G | -6.48E-02 | 8.23E-04 | -6.65E-04 | 2.40E-07 | 7.20E-09 | *ZFP36L1* |
|  | rs12950549 | Chr17:42344576 | G/A | -9.62E-02 | 5.41E-07 | 4.94E-04 | 1.60E-04 | 1.24E-09 | *STAT3* |
| IBS-PD | rs2286879 | Chr4:15739257 | G/A | 6.00E-02 | 4.15E-04 | -1.04E-01 | 1.16E-07 | 1.22E-09 | *BST1* |
|  | rs34679758 | Chr16:71961528 | G/A | -8.71E-02 | 5.80E-04 | -1.28E-01 | 8.72E-07 | 1.34E-08 | *PKD1L3* |

SNP: single nucleotide polymorphism; Chr: chromosome; Beta: the estimated effect size of cross-trait meta-analysis; *p*_CPASSOC:_ *p* value of cross-trait meta-analysis; Gene: 3D interacting genes of index SNPs were mapped by using 3DSNP; CD: Crohn's disease; UC: ulcerative colitis; IBS: irritable bowel syndrome; PD: Parkinson's disease; MS: multiple sclerosis.

**Supplementary Table 7. Novel pleiotropic loci between intestinal and neurological diseases in the MHC region (*p*_CPASSOC_<5×10^-8^, 5×10^-8^<*p*_single-trait_<1×10^-3^).**

| **Traits** | **SNP** | **Chr: Position** | **A1** | **A2** | **Trait 1** | | **Trait 2** | | ***p*_CPASSOC_** | **Gene** |
| --- | --- | --- | --- | --- | --- | --- | --- | --- | --- | --- |
|  |  |  |  |  | **Beta** | ***p*** | **Beta** | ***p*** |  |  |
| CD-MS | rs3128906 | Chr6: 29861819 | G | A | 1.20E-01 | 3.16E-04 | -7.99E-04 | 9.40E-08 | 6.64E-10 | *HCG4* |
|  | rs2517582 | Chr6: 30840985 | T | C | -1.66E-01 | 3.37E-07 | -5.98E-04 | 3.20E-06 | 1.68E-11 | *LINC02570* |
|  | rs3095303 | Chr6: 31102301 | A | G | -1.76E-01 | 1.27E-07 | 5.38E-04 | 6.00E-05 | 2.85E-10 | *C6orf15* |
|  | rs2524082 | Chr6: 31273984 | T | A | 1.68E-01 | 1.01E-07 | -6.38E-04 | 4.00E-07 | 8.51E-13 | *HLA-C* |
|  | rs9266061 | Chr6: 31351511 | G | A | 1.59E-01 | 2.66E-05 | -5.91E-04 | 1.30E-05 | 5.67E-09 | *HCG27* |
|  | rs2844503 | Chr6: 31474954 | A | T | -1.42E-01 | 6.77E-06 | -5.86E-04 | 3.30E-06 | 2.86E-10 | *MICB* |
|  | rs3132454 | Chr6: 31521867 | G | A | 1.35E-01 | 4.51E-05 | -5.43E-04 | 3.70E-05 | 2.71E-08 | *SNORD117* |
|  | rs2516050 | Chr6: 32602575 | A | G | 1.59E-01 | 4.24E-07 | -6.78E-04 | 1.40E-07 | 1.06E-12 | *HLA-DQA1* |
|  | rs9272309 | Chr6: 32636159 | G | A | 1.75E-01 | 1.13E-06 | -5.50E-04 | 2.10E-05 | 5.51E-10 | *HLA-DQA1* |
|  | rs9277895 | Chr6: 33147829 | G | A | -1.12E-01 | 6.72E-04 | -6.31E-04 | 1.80E-06 | 1.77E-08 | *HCG24* |
| CD-PD | rs34553045 | Chr6: 32593788 | C | T | -1.67E-01 | 2.31E-04 | -2.95E-01 | 1.05E-07 | 7.31E-10 | *HLA-DQA1* |
|  | rs141454996 | Chr6: 32797719 | A | C | 3.62E-01 | 1.58E-05 | 1.29E+00 | 3.24E-05 | 1.38E-08 | *HLA-DQB2* |
| UC-MS | rs1736919 | Chr6: 29729740 | G | A | 6.66E-02 | 5.02E-04 | -6.95E-04 | 3.50E-07 | 1.60E-09 | *HLA-F* |
|  | rs28400887 | Chr6: 30108126 | T | C | 8.90E-02 | 7.65E-06 | -7.71E-04 | 1.20E-07 | 5.39E-12 | *TRIM31* |
|  | rs1265108 | Chr6: 31152092 | T | C | -9.92E-02 | 1.05E-07 | -6.94E-04 | 2.10E-07 | 8.88E-13 | *CCHCR1* |
|  | rs9266716 | Chr6: 31381950 | T | C | -9.26E-02 | 9.36E-06 | -6.88E-04 | 3.50E-07 | 9.55E-11 | *HLA-B* |
|  | rs2596464 | Chr6: 31445184 | C | T | 7.19E-02 | 1.41E-04 | -5.39E-04 | 1.70E-05 | 1.69E-08 | *LINC01149* |
|  | rs3134928 | Chr6: 32225787 | T | C | -7.75E-02 | 2.79E-04 | -7.71E-04 | 2.00E-07 | 1.82E-09 | *NOTCH4* |
|  | rs9271692 | Chr6: 32625233 | A | C | 6.62E-02 | 3.69E-04 | -6.61E-04 | 3.00E-07 | 9.52E-10 | *HLA-DQA1* |
|  | rs2894381 | Chr6: 32714451 | A | G | 7.72E-02 | 8.69E-04 | -7.68E-04 | 7.40E-07 | 6.14E-09 | *HLA-DQA1* |
|  | rs194675 | Chr6: 32937969 | A | T | -6.94E-02 | 2.36E-04 | -5.82E-04 | 5.00E-06 | 2.89E-08 | *HLA-DMB* |
|  | rs3130580 | Chr6: 33051305 | C | G | -1.10E-01 | 2.93E-05 | -8.25E-04 | 6.80E-08 | 6.42E-11 | *HLA-DPA1* |
|  | rs2213309 | Chr6: 33077478 | C | T | -6.96E-02 | 1.93E-04 | -6.60E-04 | 1.90E-07 | 1.20E-09 | *HLA-DPA1* |
| UC-PD | rs9268627 | Chr6: 32438044 | C | T | -1.18E-01 | 1.12E-06 | -1.28E-01 | 4.77E-07 | 2.07E-12 | *HLA-DRA* |
| IBS-MS | rs2524227 | Chr6: 31334786 | C | T | 5.20E-02 | 8.22E-04 | -6.23E-04 | 1.40E-06 | 1.40E-08 | *HLA-C* |

SNP: single nucleotide polymorphism; Chr: chromosome; Beta: the estimated effect size of cross-trait meta-analysis; *p*_CPASSOC:_ *p* value of cross-trait meta-analysis; Gene: 3D interacting genes of index SNPs were mapped by using 3DSNP; CD: Crohn's disease; UC: ulcerative colitis; IBS: irritable bowel syndrome; MS: multiple sclerosis; PD: Parkinson's disease.

**Supplementary Table 8. Summary of functional genes for CD, UC, PD, and MS from SMR.**

| **Trait** | **probeID** | **Chr** | **Gene** | **Position** | **SNP** | **A1** | **A2** | **b_SMR** | **SE_SMR** | ***P*_SMR** | ***P*_HEIDI** |
| --- | --- | --- | --- | --- | --- | --- | --- | --- | --- | --- | --- |
| **CD** | ILMN_2066060 | 6 | *HLA-DRB6* | 32520619 | rs9270529 | T | C | 0.199 | 0.037 | 6.94E-08 | 6.31E-04 |
|  | ILMN_2066066 | 6 | *HLA-DRB6* | 32521719 | rs35139284 | T | C | 0.619 | 0.094 | 4.41E-11 | 1.41E-02 |
|  | ILMN_1715169 | 6 | *HLA-DRB1* | 32548032 | rs9271179 | G | A | -0.153 | 0.030 | 2.35E-07 | 1.95E-04 |
|  | ILMN_1808707 | 7 | *FSCN1* | 5646166 | rs4415230 | T | C | -0.496 | 0.105 | 2.17E-06 | 1.38E-08 |
| **UC** | ILMN_1744713 | 1 | *PARK7* | 8045070 | rs35675666 | T | G | -0.244 | 0.050 | 1.39E-06 | 1.49E-03 |
|  | ILMN_1658383 | 1 | *HSPA7* | 161577056 | rs2099684 | G | A | -0.201 | 0.044 | 4.44E-06 | 3.82E-03 |
|  | ILMN_1806165 | 1 | *HSPA7* | 161578021 | rs10919544 | C | T | -0.239 | 0.048 | 5.90E-07 | 8.11E-04 |
|  | ILMN_1662524 | 2 | *CXCR1* | 219027921 | rs13397673 | G | A | -0.192 | 0.042 | 4.73E-06 | 8.18E-02 |
|  | ILMN_1718023 | 3 | *APEH* | 49720758 | rs1131095 | C | T | 0.560 | 0.074 | 3.10E-14 | 1.96E-02 |
|  | ILMN_1690454 | 3 | *C3orf54* | 49842332 | rs6802890 | G | A | 0.713 | 0.152 | 2.59E-06 | 3.24E-02 |
|  | ILMN_1794612 | 3 | *UBA7* | 49842691 | rs6775384 | A | G | 0.180 | 0.028 | 1.23E-10 | 7.39E-06 |
|  | ILMN_1666078 | 6 | *HLA-H* | 29857600 | rs2735059 | A | G | 0.124 | 0.024 | 1.91E-07 | 3.20E-02 |
|  | ILMN_2150787 | 6 | *HLA-C* | 31236618 | rs2523578 | G | A | 0.193 | 0.041 | 2.79E-06 | 5.73E-03 |
|  | ILMN_1697499 | 6 | *HLA-DRB1* | 32485422 | rs9271152 | T | G | 0.137 | 0.019 | 9.54E-13 | 2.12E-04 |
|  | ILMN_1671378 | 6 | *HLA-DRB1* | 32519520 | rs28366303 | G | C | 0.576 | 0.067 | 4.92E-18 | 3.31E-03 |
|  | ILMN_2066066 | 6 | *HLA-DRB6* | 32521719 | rs35139284 | T | C | 0.290 | 0.053 | 3.68E-08 | 4.52E-14 |
|  | ILMN_1715169 | 6 | *HLA-DRB1* | 32548032 | rs9271179 | G | A | 0.106 | 0.017 | 3.84E-10 | 1.83E-09 |
|  | ILMN_1808405 | 6 | *HLA-DQA1* | 32609840 | rs679242 | T | G | -0.218 | 0.025 | 4.79E-18 | 1.22E-16 |
|  | ILMN_1725170 | 6 | *CA425595* | 32624021 | rs9274579 | A | G | 0.172 | 0.019 | 5.62E-19 | 2.38E-01 |
|  | ILMN_1670757 | 6 | *HLA-DQB1* | 32626588 | rs4959030 | A | G | 0.780 | 0.158 | 7.89E-07 | 1.88E-02 |
|  | ILMN_1808707 | 7 | *FSCN1* | 5646166 | rs4415230 | T | C | -0.581 | 0.080 | 4.30E-13 | 6.53E-11 |
|  | ILMN_2300695 | 17 | *IKZF3* | 37921755 | rs907091 | T | C | 0.174 | 0.034 | 2.91E-07 | 3.28E-04 |
|  | ILMN_1666206 | 17 | *GSDMB* | 38060997 | rs12936231 | C | G | -0.104 | 0.020 | 2.75E-07 | 2.81E-04 |
|  | ILMN_2347193 | 17 | *GSDMB* | 38062136 | rs12936231 | C | G | -0.128 | 0.025 | 3.28E-07 | 7.57E-05 |
|  | ILMN_2260756 | 17 | *GSDMB* | 38073727 | rs7216558 | C | T | -0.269 | 0.056 | 1.45E-06 | 1.66E-03 |
|  | ILMN_1662174 | 17 | *ORMDL3* | 38077572 | rs12936231 | C | G | -0.121 | 0.024 | 3.11E-07 | 2.73E-04 |
|  | ILMN_2183687 | 20 | *LIME1* | 62370429 | rs6062509 | G | T | -0.205 | 0.044 | 4.09E-06 | 1.29E-03 |
| **PD** | ILMN_1751500 | 1 | *ADAM15* | 155032795 | rs11264304 | T | C | 0.167 | 0.033 | 3.06E-07 | 5.51E-05 |
|  | ILMN_1813685 | 1 | *RAB7L1* | 205738399 | rs708723 | C | T | 0.335 | 0.066 | 4.44E-07 | 5.45E-03 |
|  | ILMN_2342835 | 3 | *P2RY14* | 150931328 | rs111558233 | T | C | -0.200 | 0.043 | 2.92E-06 | 8.52E-01 |
|  | ILMN_1654629 | 4 | *TMEM175* | 952381 | rs6599388 | T | C | -1.003 | 0.198 | 4.20E-07 | 4.37E-04 |
|  | ILMN_1793017 | 4 | *DGKQ* | 952839 | rs1377586 | A | G | -0.095 | 0.017 | 1.61E-08 | 2.25E-04 |
|  | ILMN_1703041 | 4 | *IDUA* | 997408 | rs13101828 | G | A | -0.212 | 0.041 | 2.53E-07 | 2.26E-03 |
|  | ILMN_1770161 | 4 | *BST1* | 15733490 | rs3213710 | G | A | -0.189 | 0.028 | 1.79E-11 | 2.72E-01 |
|  | ILMN_1701933 | 4 | *SNCA* | 90647613 | rs1372520 | T | C | -0.429 | 0.048 | 7.41E-19 | 2.58E-10 |
|  | ILMN_1766165 | 4 | *SNCA* | 90650397 | rs1372520 | T | C | -0.428 | 0.048 | 5.60E-19 | 2.02E-11 |
|  | ILMN_1808405 | 6 | *HLA-DQA1* | 32609840 | rs532965 | G | T | -0.163 | 0.026 | 4.61E-10 | 2.01E-04 |
|  | ILMN_1789616 | 7 | *NUPL2* | 23240132 | rs858300 | G | A | 0.337 | 0.065 | 2.25E-07 | 3.74E-01 |
|  | ILMN_2115154 | 7 | *NUPL2* | 23240217 | rs1637221 | C | T | 0.276 | 0.051 | 8.04E-08 | 4.06E-02 |
|  | ILMN_1680313 | 16 | *STX4* | 31051363 | rs9673641 | A | G | -0.277 | 0.060 | 4.10E-06 | 9.77E-04 |
|  | ILMN_1739236 | 16 | *ZNF668* | 31072283 | rs2303222 | C | T | 0.234 | 0.051 | 4.74E-06 | 3.14E-04 |
|  | ILMN_1693394 | 16 | *BCKDK* | 31123838 | rs12716982 | A | C | 0.348 | 0.073 | 1.86E-06 | 5.00E-03 |
|  | ILMN_1703946 | 17 | *ADORA2B* | 15878773 | rs2159643 | C | G | 0.227 | 0.047 | 1.34E-06 | 1.48E-01 |
|  | ILMN_2192385 | 17 | *TTC19* | 15931706 | rs2301652 | T | G | -0.202 | 0.043 | 2.91E-06 | 1.02E-01 |
|  | ILMN_2393693 | 17 | *LRRC37A4* | 43584508 | rs113661667 | C | T | 0.161 | 0.021 | 2.30E-14 | 2.40E-06 |
|  | ILMN_2286783 | 17 | *LRRC37A4* | 43585603 | rs55668363 | A | G | 1.170 | 0.230 | 3.81E-07 | 1.60E-02 |
|  | ILMN_1785277 | 17 | *DB316129* | 43602326 | rs112073200 | C | G | -1.134 | 0.218 | 2.01E-07 | 7.31E-03 |
|  | ILMN_1784428 | 17 | *MGC57346* | 43715150 | rs62057067 | T | C | -0.261 | 0.030 | 1.14E-18 | 4.11E-05 |
|  | ILMN_1743621 | 17 | *C17orf69* | 43719529 | rs113029914 | T | A | -0.580 | 0.079 | 1.65E-13 | 2.47E-05 |
|  | ILMN_1783673 | 17 | *LRRC37A2* | 44626174 | rs112431991 | G | A | -1.162 | 0.226 | 2.57E-07 | 4.76E-03 |
|  | ILMN_2330845 | 17 | *NSF* | 44834256 | rs199446 | G | A | -1.025 | 0.195 | 1.39E-07 | 7.25E-04 |
|  | ILMN_1680353 | 17 | *NSF* | 44834656 | rs199442 | A | G | -0.853 | 0.143 | 2.20E-09 | 1.08E-03 |
| **MS** | ILMN_2186806 | 6 | *HLA-A* | 29693978 | rs2471863 | T | C | -0.002 | 0.000 | 4.86E-19 | 6.18E-05 |
|  | ILMN_1660923 | 6 | *NA* | 29760774 | rs2471863 | T | C | -0.002 | 0.000 | 4.63E-20 | 1.16E-03 |
|  | ILMN_1666078 | 6 | *HLA-H* | 29857600 | rs2471863 | T | C | -0.001 | 0.000 | 2.22E-20 | 1.17E-03 |
|  | ILMN_2130441 | 6 | *HLA-H* | 29858241 | rs2471863 | T | C | -0.002 | 0.000 | 9.66E-19 | 5.09E-04 |
|  | ILMN_1749409 | 6 | *HLA-F* | 29976886 | rs2517910 | A | C | 0.003 | 0.000 | 3.43E-08 | 1.13E-03 |
|  | ILMN_2150787 | 6 | *HLA-C* | 31236618 | rs2523578 | G | A | 0.002 | 0.000 | 1.16E-16 | 1.13E-11 |
|  | ILMN_1654541 | 6 | *ATP6V1G2* | 31512600 | rs2395042 | A | C | -0.004 | 0.001 | 1.15E-14 | 8.54E-03 |
|  | ILMN_1660436 | 6 | *HSPA1A* | 31797709 | rs494620 | A | G | -0.002 | 0.000 | 9.27E-15 | 1.10E-12 |
|  | ILMN_1773082 | 6 | *CYP21A1P* | 31976517 | rs3132463 | T | C | -0.008 | 0.002 | 2.53E-07 | 1.22E-02 |
|  | ILMN_1679520 | 6 | *AGPAT1* | 32136453 | rs553108 | A | G | -0.011 | 0.002 | 5.01E-07 | 5.41E-03 |
|  | ILMN_1697499 | 6 | *HLA-DRB1* | 32485422 | rs9271116 | T | G | 0.003 | 0.000 | 2.04E-70 | 5.30E-04 |
|  | ILMN_1671378 | 6 | *HLA-DRB1* | 32519520 | rs28366302 | C | G | 0.004 | 0.000 | 2.33E-19 | 1.00E-04 |
|  | ILMN_2066066 | 6 | *HLA-DRB6* | 32521719 | rs9270588 | C | T | -0.003 | 0.000 | 6.79E-13 | 8.71E-14 |
|  | ILMN_1715169 | 6 | *HLA-DRB1* | 32548032 | rs9271178 | C | T | 0.002 | 0.000 | 2.75E-52 | 9.87E-21 |
|  | ILMN_1725170 | 6 | *CA425595* | 32624021 | rs3828789 | G | T | 0.001 | 0.000 | 2.02E-25 | 3.27E-17 |
|  | ILMN_1670757 | 6 | *HLA-DQB1* | 32626588 | rs4286817 | A | G | 0.011 | 0.002 | 1.19E-07 | 5.16E-02 |
|  | ILMN_1695311 | 6 | *HLA-DMA* | 32916436 | rs9271116 | T | G | 0.009 | 0.001 | 1.54E-20 | 3.55E-03 |
|  | ILMN_1791006 | 6 | *AHI1* | 135605307 | rs6908428 | G | A | -0.001 | 0.000 | 1.13E-07 | 4.56E-01 |

CD: Crohn's disease; UC: ulcerative colitis; MS: multiple sclerosis; PD: Parkinson's disease; Chr: chromosome; SE: standard error.

**Supplementary Table 9. Summary of functional genes for CD, UC, PD, and MS after HEIDE test.**

| **Trait** | **probeID** | **Chr** | **Gene** | **Position** | **SNP** | **A1** | **A2** | **b_SMR** | **SE_SMR** | ***P*_SMR** | ***P*_HEIDI** |
| --- | --- | --- | --- | --- | --- | --- | --- | --- | --- | --- | --- |
| **CD** | ILMN_2066066 | 6 | *HLA-DRB6* | 32521719 | rs35139284 | T | C | 0.619 | 0.094 | 4.41E-11 | 1.41E-02 |
| **UC** | ILMN_1662524 | 2 | *CXCR1* | 219027921 | rs13397673 | G | A | -0.192 | 0.042 | 4.73E-06 | 8.18E-02 |
|  | ILMN_1718023 | 3 | *APEH* | 49720758 | rs1131095 | C | T | 0.560 | 0.074 | 3.10E-14 | 1.96E-02 |
|  | ILMN_1690454 | 3 | *C3orf54* | 49842332 | rs6802890 | G | A | 0.713 | 0.152 | 2.59E-06 | 3.24E-02 |
|  | ILMN_1666078 | 6 | *HLA-H* | 29857600 | rs2735059 | A | G | 0.124 | 0.024 | 1.91E-07 | 3.20E-02 |
|  | ILMN_1725170 | 6 | *CA425595* | 32624021 | rs9274579 | A | G | 0.172 | 0.019 | 5.62E-19 | 2.38E-01 |
|  | ILMN_1670757 | 6 | *HLA-DQB1* | 32626588 | rs4959030 | A | G | 0.780 | 0.158 | 7.89E-07 | 1.88E-02 |
| **PD** | ILMN_2342835 | 3 | *P2RY14* | 150931328 | rs111558233 | T | C | -0.200 | 0.043 | 2.92E-06 | 8.52E-01 |
|  | ILMN_1770161 | 4 | *BST1* | 15733490 | rs3213710 | G | A | -0.189 | 0.028 | 1.79E-11 | 2.72E-01 |
|  | ILMN_1789616 | 7 | *NUPL2* | 23240132 | rs858300 | G | A | 0.337 | 0.065 | 2.25E-07 | 3.74E-01 |
|  | ILMN_2115154 | 7 | *NUPL2* | 23240217 | rs1637221 | C | T | 0.276 | 0.051 | 8.04E-08 | 4.06E-02 |
|  | ILMN_1703946 | 17 | *ADORA2B* | 15878773 | rs2159643 | C | G | 0.227 | 0.047 | 1.34E-06 | 1.48E-01 |
|  | ILMN_2192385 | 17 | *TTC19* | 15931706 | rs2301652 | T | G | -0.202 | 0.043 | 2.91E-06 | 1.02E-01 |
|  | ILMN_2286783 | 17 | *LRRC37A4* | 43585603 | rs55668363 | A | G | 1.170 | 0.230 | 3.81E-07 | 1.60E-02 |
| **MS** | ILMN_1773082 | 6 | *CYP21A1P* | 31976517 | rs3132463 | T | C | -0.008 | 0.002 | 2.53E-07 | 1.22E-02 |
|  | ILMN_1670757 | 6 | *HLA-DQB1* | 32626588 | rs4286817 | A | G | 0.011 | 0.002 | 1.19E-07 | 5.16E-02 |
|  | ILMN_1791006 | 6 | *AHI1* | 135605307 | rs6908428 | G | A | -0.001 | 0.000 | 1.13E-07 | 4.56E-01 |

CD: Crohn's disease; UC: ulcerative colitis; MS: multiple sclerosis; PD: Parkinson's disease; Chr: chromosome; SE: standard error.

**Supplementary Table 10. Functional genes shared between intestinal and neurological disease based on SMR.**

| **Phenotypes** | **Gene** | **probeID** | **ProbeChr** | **Probe_bp** | **topSNP** | **A1** | **A2** | ***P*_GWAS** | ***P*_eQTL** | ***P*_SMR** | ***P*_HEIDI** |
| --- | --- | --- | --- | --- | --- | --- | --- | --- | --- | --- | --- |
| UC-MS | *HLA-DQB1* | ILMN_1670757 | 6 | 32626588 | rs4959030 | A | G | 5.06E-14 | 6.12E-11 | 7.89E-07 | 1.88E-02 |
|  | *HLA-DQB1* | ILMN_1670757 | 6 | 32626588 | rs4286817 | A | G | 7.50E-53 | 1.67E-08 | 1.19E-07 | 5.16E-02 |

UC: ulcerative colitis; MS: multiple sclerosis.

**Supplementary table 11. Summary of SNP heritability enrichment among for chromatin data from the Roadmap Epigenomics and ENCODE.**

| **Trait** | **Name** | **Coefficient** | **Coefficient_SE** | **Coefficient_*P*_value** | ***P*_adjust** |
| --- | --- | --- | --- | --- | --- |
| UC | Colonic_Mucosa__H3K27ac | 8.34E-08 | 2.65E-08 | 8.30E-04 | 2.77E-02 |
|  | Colonic_Mucosa__H3K4me1 | 5.18E-08 | 1.42E-08 | 1.37E-04 | 1.62E-02 |
|  | Colonic_Mucosa__H3K9ac | 1.73E-07 | 5.53E-08 | 8.89E-04 | 2.77E-02 |
|  | Primary_Natural_Killer_cells_from_peripheral_blood__DNase | 6.12E-08 | 2.01E-08 | 1.14E-03 | 2.94E-02 |
|  | Primary_Natural_Killer_cells_from_peripheral_blood__H3K4me1 | 3.39E-08 | 9.30E-09 | 1.35E-04 | 1.62E-02 |
|  | Primary_T_cells_from_peripheral_blood__H3K4me1 | 3.19E-08 | 9.06E-09 | 2.13E-04 | 1.62E-02 |
|  | Primary_T_helper_17_cells_PMA-I_stimulated__H3K27ac | 5.06E-08 | 1.65E-08 | 1.08E-03 | 2.93E-02 |
|  | Primary_T_helper_17_cells_PMA-I_stimulated__H3K4me1 | 4.10E-08 | 9.91E-09 | 1.73E-05 | 8.46E-03 |
|  | Primary_T_helper_cells_from_peripheral_blood__H3K4me1 | 2.46E-08 | 7.39E-09 | 4.31E-04 | 2.11E-02 |
|  | Primary_T_helper_cells_PMA-I_stimulated__H3K4me1 | 2.03E-08 | 6.41E-09 | 7.76E-04 | 2.77E-02 |
|  | Primary_T_helper_memory_cells_from_peripheral_blood_1__H3K4me1 | 2.48E-08 | 8.04E-09 | 1.02E-03 | 2.92E-02 |
|  | Primary_T_helper_memory_cells_from_peripheral_blood_2__H3K4me1 | 2.50E-08 | 7.45E-09 | 3.95E-04 | 2.11E-02 |
|  | Primary_T_regulatory_cells_from_peripheral_blood__H3K4me1 | 2.59E-08 | 8.91E-09 | 1.82E-03 | 4.23E-02 |
|  | Rectal_Mucosa_Donor_29__H3K27ac | 6.96E-08 | 2.08E-08 | 3.99E-04 | 2.11E-02 |
|  | Rectal_Mucosa_Donor_29__H3K9ac | 1.65E-07 | 5.02E-08 | 5.19E-04 | 2.31E-02 |
|  | Rectal_Mucosa_Donor_31__H3K27ac | 5.55E-08 | 1.89E-08 | 1.67E-03 | 4.09E-02 |
|  | Rectal_Mucosa_Donor_31__H3K9ac | 1.01E-07 | 3.19E-08 | 7.99E-04 | 2.77E-02 |
|  | Sigmoid_Colon__H3K27ac | 9.02E-08 | 2.53E-08 | 1.79E-04 | 1.62E-02 |
|  | SI-Term-Ileum_ENTEX__H3K27ac | 3.67E-08 | 1.05E-08 | 2.32E-04 | 1.62E-02 |
|  | Small_Intestine__H3K27ac | 9.74E-08 | 2.72E-08 | 1.70E-04 | 1.62E-02 |
|  | Spleen_ENTEX__H3K4me1 | 7.21E-08 | 2.31E-08 | 9.06E-04 | 2.77E-02 |
| MS | Primary_T_helper_17_cells_PMA-I_stimulated__H3K4me1 | 1.68E-08 | 4.90E-09 | 3.04E-04 | 3.99E-02 |
|  | Primary_T_helper_cells_from_peripheral_blood__H3K4me1 | 1.65E-08 | 4.84E-09 | 3.26E-04 | 3.99E-02 |
|  | Primary_T_helper_cells_PMA-I_stimulated__H3K27ac | 1.63E-08 | 4.94E-09 | 5.07E-04 | 4.44E-02 |
|  | Primary_T_helper_cells_PMA-I_stimulated__H3K4me1 | 1.56E-08 | 4.23E-09 | 1.07E-04 | 3.99E-02 |
|  | Primary_T_helper_memory_cells_from_peripheral_blood_1__H3K4me1 | 1.44E-08 | 4.46E-09 | 6.35E-04 | 4.44E-02 |
|  | Primary_T_helper_memory_cells_from_peripheral_blood_2__H3K4me1 | 1.51E-08 | 4.27E-09 | 2.04E-04 | 3.99E-02 |
|  | Primary_T_helper_naive_cells_from_peripheral_blood_1__H3K4me1 | 1.74E-08 | 5.52E-09 | 8.13E-04 | 4.97E-02 |
|  | Primary_T_killer_naive_cells_from_peripheral_blood__H3K4me1 | 1.59E-08 | 4.93E-09 | 6.20E-04 | 4.44E-02 |

UC: ulcerative colitis; MS: multiple sclerosis.

**Supplementary Table 12. Causal relationship between CD and PD based on MR methods.**

| exposure | outcome | method | IV | Beta | SE | *p* | OR | OR_lci95 | OR_uci95 | FDR |
| --- | --- | --- | --- | --- | --- | --- | --- | --- | --- | --- |
| CD | PD | MR Egger | 13 | 0.067 | 0.094 | 4.88E-01 | 1.069 | 0.890 | 1.285 | 4.88E-01 |
|  |  | Weighted median | 13 | 0.098 | 0.04 | 1.40E-02 | 1.103 | 1.020 | 1.192 | 3.51E-02 |
|  |  | Inverse variance weighted | 13 | 0.088 | 0.029 | 2.68E-03 | 1.092 | 1.031 | 1.156 | 1.34E-02 |
|  |  | Simple mode | 13 | 0.113 | 0.066 | 1.15E-01 | 1.119 | 0.983 | 1.275 | 1.44E-01 |
|  |  | Weighted mode | 13 | 0.099 | 0.057 | 1.07E-01 | 1.104 | 0.988 | 1.235 | 1.44E-01 |

CD: Crohn's disease; PD: Parkinson's disease; IV: instrumental variables; Beta: beta value; SE: standard error; CI: confidence interval; OR: odd ratio; FDR: False discovery rate.

**Supplementary table 13. Causal relationship between trait-associated CpG loci and diseases based on MR methods**

| **Exposure** | | **Outcome** | **Method** | **IV** | **Beta** | **SE** | ***P*** | **lo_ci** | **up_ci** | **OR** | **OR_lci95** | **OR_uci95** | **FDR** |
| --- | --- | --- | --- | --- | --- | --- | --- | --- | --- | --- | --- | --- | --- |
| UC | cg25114611 | PD | MR Egger | 4 | 4.73E-02 | 1.76E-01 | 8.13E-01 | -2.97E-01 | 3.92E-01 | 1.05E+00 | 7.43E-01 | 1.48E+00 | 8.13E-01 |
|  |  |  | Weighted median | 4 | -1.99E-01 | 7.82E-02 | 1.08E-02 | -3.52E-01 | -4.60E-02 | 8.19E-01 | 7.03E-01 | 9.55E-01 | 4.83E-02 |
|  |  |  | Inverse variance weighted | 4 | -1.54E-01 | 6.59E-02 | 1.93E-02 | -2.83E-01 | -2.50E-02 | 8.57E-01 | 7.53E-01 | 9.75E-01 | 4.83E-02 |
|  |  |  | Simple mode | 4 | -2.10E-01 | 1.07E-01 | 1.44E-01 | -4.19E-01 | -8.07E-04 | 8.10E-01 | 6.57E-01 | 9.99E-01 | 1.80E-01 |
|  |  |  | Weighted mode | 4 | -2.09E-01 | 1.02E-01 | 1.34E-01 | -4.09E-01 | -7.87E-03 | 8.12E-01 | 6.64E-01 | 9.92E-01 | 1.80E-01 |
| AD | cg13455960 | CD | MR Egger | 10 | 3.47E-01 | 1.53E-01 | 5.38E-02 | 4.59E-02 | 6.47E-01 | 1.41E+00 | 1.05E+00 | 1.91E+00 | 5.38E-02 |
|  |  |  | Weighted median | 10 | 2.92E-01 | 8.23E-02 | 3.90E-04 | 1.31E-01 | 4.53E-01 | 1.34E+00 | 1.14E+00 | 1.57E+00 | 9.74E-04 |
|  |  |  | Inverse variance weighted | 10 | 2.43E-01 | 6.06E-02 | 6.18E-05 | 1.24E-01 | 3.61E-01 | 1.27E+00 | 1.13E+00 | 1.44E+00 | 3.09E-04 |
|  |  |  | Simple mode | 10 | 2.66E-01 | 1.19E-01 | 5.15E-02 | 3.36E-02 | 4.98E-01 | 1.30E+00 | 1.03E+00 | 1.65E+00 | 5.38E-02 |
|  |  |  | Weighted mode | 10 | 2.73E-01 | 9.45E-02 | 1.80E-02 | 8.74E-02 | 4.58E-01 | 1.31E+00 | 1.09E+00 | 1.58E+00 | 3.00E-02 |
|  | cg19240213 | CD | MR Egger | 19 | -1.14E-01 | 8.22E-02 | 1.82E-01 | -2.76E-01 | 4.66E-02 | 8.92E-01 | 7.59E-01 | 1.05E+00 | 1.82E-01 |
|  |  |  | Weighted median | 19 | -1.78E-01 | 5.18E-02 | 6.04E-04 | -2.79E-01 | -7.61E-02 | 8.37E-01 | 7.56E-01 | 9.27E-01 | 1.51E-03 |
|  |  |  | Inverse variance weighted | 19 | -1.56E-01 | 3.64E-02 | 1.89E-05 | -2.27E-01 | -8.44E-02 | 8.56E-01 | 7.97E-01 | 9.19E-01 | 9.45E-05 |
|  |  |  | Simple mode | 19 | -1.87E-01 | 8.49E-02 | 4.08E-02 | -3.53E-01 | -2.07E-02 | 8.29E-01 | 7.02E-01 | 9.80E-01 | 5.11E-02 |
|  |  |  | Weighted mode | 19 | -1.57E-01 | 5.03E-02 | 5.96E-03 | -2.55E-01 | -5.82E-02 | 8.55E-01 | 7.75E-01 | 9.43E-01 | 9.94E-03 |
|  | cg06889108 | UC | MR Egger | 6 | 5.37E-02 | 4.34E-02 | 2.84E-01 | -3.14E-02 | 1.39E-01 | 1.06E+00 | 9.69E-01 | 1.15E+00 | 2.84E-01 |
|  |  |  | Weighted median | 6 | 6.71E-02 | 2.52E-02 | 7.68E-03 | 1.78E-02 | 1.16E-01 | 1.07E+00 | 1.02E+00 | 1.12E+00 | 2.06E-02 |
|  |  |  | Inverse variance weighted | 6 | 6.33E-02 | 2.39E-02 | 8.23E-03 | 1.63E-02 | 1.10E-01 | 1.07E+00 | 1.02E+00 | 1.12E+00 | 2.06E-02 |
|  |  |  | Simple mode | 6 | 9.78E-02 | 4.42E-02 | 7.79E-02 | 1.11E-02 | 1.85E-01 | 1.10E+00 | 1.01E+00 | 1.20E+00 | 1.30E-01 |
|  |  |  | Weighted mode | 6 | 5.47E-02 | 2.86E-02 | 1.14E-01 | -1.39E-03 | 1.11E-01 | 1.06E+00 | 9.99E-01 | 1.12E+00 | 1.43E-01 |
| Epilepsy | cg02276823 | CD | MR Egger | 17 | -2.00E-02 | 8.32E-02 | 8.14E-01 | -1.83E-01 | 1.43E-01 | 9.80E-01 | 8.33E-01 | 1.15E+00 | 8.14E-01 |
|  |  |  | Weighted median | 17 | 1.18E-01 | 5.77E-02 | 4.14E-02 | 4.57E-03 | 2.31E-01 | 1.12E+00 | 1.00E+00 | 1.26E+00 | 1.04E-01 |
|  |  |  | Inverse variance weighted | 17 | 1.75E-01 | 4.71E-02 | 2.01E-04 | 8.29E-02 | 2.68E-01 | 1.19E+00 | 1.09E+00 | 1.31E+00 | 1.00E-03 |
|  |  |  | Simple mode | 17 | 7.35E-02 | 8.82E-02 | 4.17E-01 | -9.93E-02 | 2.46E-01 | 1.08E+00 | 9.05E-01 | 1.28E+00 | 5.21E-01 |
|  |  |  | Weighted mode | 17 | 1.12E-01 | 6.31E-02 | 9.43E-02 | -1.14E-02 | 2.36E-01 | 1.12E+00 | 9.89E-01 | 1.27E+00 | 1.57E-01 |
|  | cg02530824 | IBS | MR Egger | 6 | 1.30E-01 | 4.93E-02 | 5.73E-02 | 3.37E-02 | 2.27E-01 | 1.14E+00 | 1.03E+00 | 1.25E+00 | 9.56E-02 |
|  |  |  | Weighted median | 6 | 8.32E-02 | 3.59E-02 | 2.06E-02 | 1.28E-02 | 1.54E-01 | 1.09E+00 | 1.01E+00 | 1.17E+00 | 5.15E-02 |
|  |  |  | Inverse variance weighted | 6 | 8.14E-02 | 3.04E-02 | 7.47E-03 | 2.18E-02 | 1.41E-01 | 1.08E+00 | 1.02E+00 | 1.15E+00 | 3.73E-02 |
|  |  |  | Simple mode | 6 | 5.40E-02 | 4.77E-02 | 3.09E-01 | -3.95E-02 | 1.48E-01 | 1.06E+00 | 9.61E-01 | 1.16E+00 | 3.09E-01 |
|  |  |  | Weighted mode | 6 | 8.58E-02 | 4.02E-02 | 8.61E-02 | 6.94E-03 | 1.65E-01 | 1.09E+00 | 1.01E+00 | 1.18E+00 | 1.08E-01 |
| Migraine | cg24138857 | IBS | MR Egger | 10 | 4.81E-02 | 1.30E-01 | 7.21E-01 | -2.07E-01 | 3.03E-01 | 1.05E+00 | 8.13E-01 | 1.35E+00 | 7.21E-01 |
|  |  |  | Weighted median | 10 | -1.38E-01 | 5.64E-02 | 1.43E-02 | -2.49E-01 | -2.76E-02 | 8.71E-01 | 7.80E-01 | 9.73E-01 | 3.58E-02 |
|  |  |  | Inverse variance weighted | 10 | -1.19E-01 | 4.31E-02 | 5.87E-03 | -2.03E-01 | -3.43E-02 | 8.88E-01 | 8.16E-01 | 9.66E-01 | 2.93E-02 |
|  |  |  | Simple mode | 10 | -1.70E-01 | 9.81E-02 | 1.17E-01 | -3.62E-01 | 2.24E-02 | 8.44E-01 | 6.96E-01 | 1.02E+00 | 1.47E-01 |
|  |  |  | Weighted mode | 10 | -1.50E-01 | 8.59E-02 | 1.15E-01 | -3.18E-01 | 1.83E-02 | 8.61E-01 | 7.27E-01 | 1.02E+00 | 1.47E-01 |

CD: Crohn's disease; UC: ulcerative colitis; IBS: irritable bowel syndrome; AD: Alzheimer's disease; PD: Parkinson's disease; IV: instrumental variables; Beta: beta value; SE: standard error; CI: confidence interval; OR: odd ratio; FDR: False discovery rate.
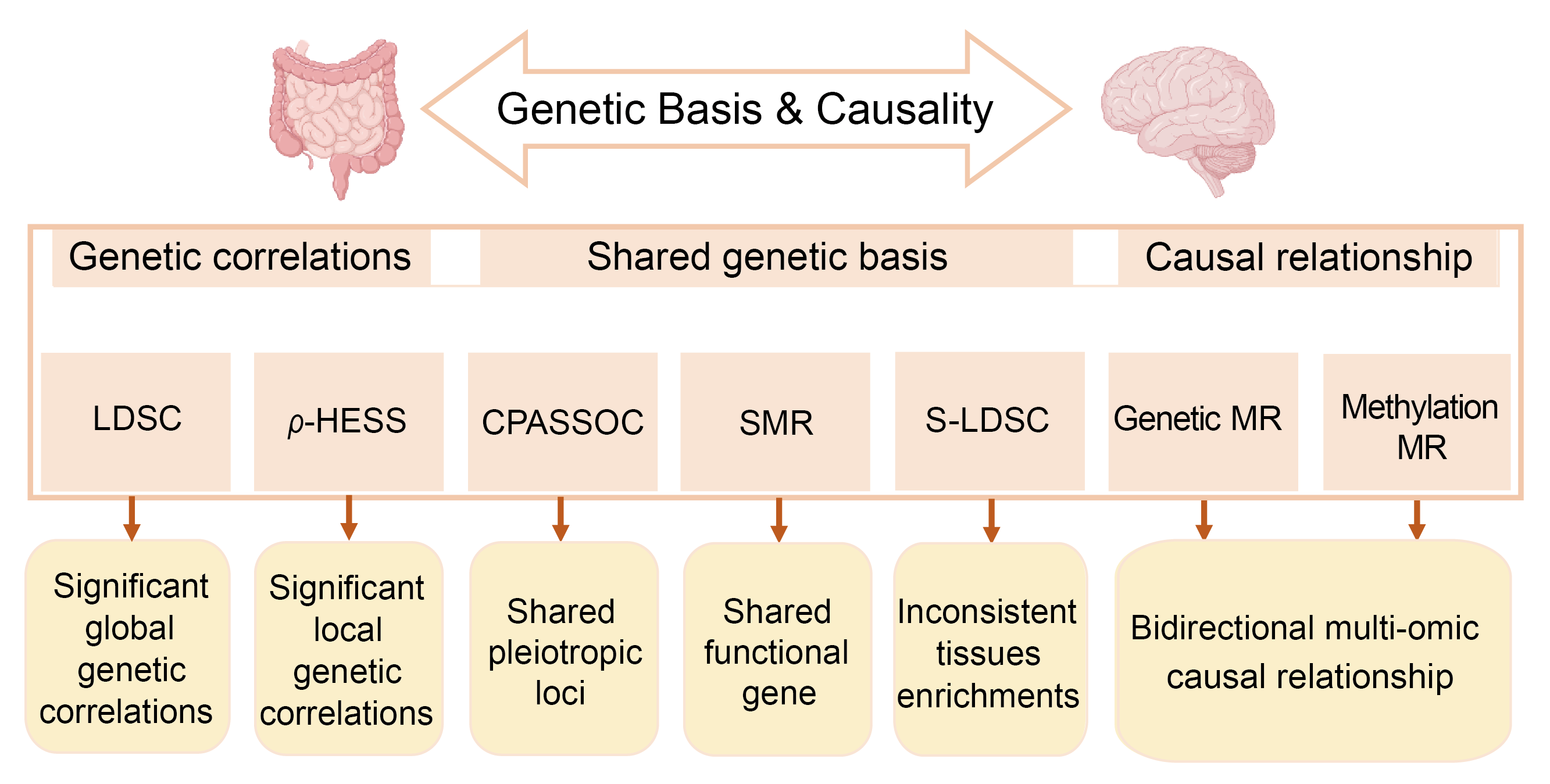


**Supplementary Figure1. Overall study design.**

This figure illustrates the analytical workflow for investigating the genetic basis and causal relationships between intestinal and neurological diseases. First, the genome-wide genetic correlations among the study traits were assessed using the LDSC method, and significant local genetic correlation regions were identified with the *ρ*-HESS approach. Based on this, pleiotropic loci shared across traits were screened using the CPASSOC method, and potential shared functional genes were further explored via the SMR method, with their tissue-specific enrichment also evaluated. Finally, the direction of causal relationships was inferred using MR. LDSC: Linkage Disequilibrium Score Regression, *ρ*-HESS: Heritability Estimation from Summary Statistics, CPASSOC: Cross-Phenotype Association, SMR: Summary-data-based Mendelian Randomization, s-LDSC: stratified-Linkage Disequilibrium Score Regression, MR: Mendelian Randomization.


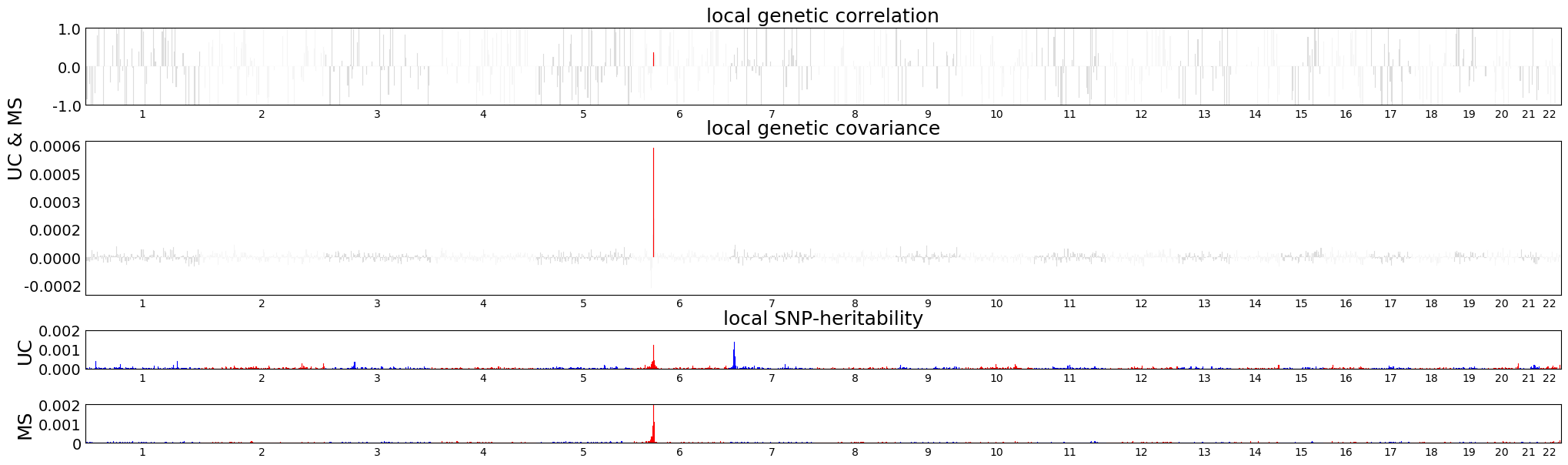
**Supplementary Figure2 Local genetic correlation between intestinal and neurological diseases**

The Manhattan plot showed estimates of local genetic correlations, genetic covariance, and SNP heritability between ulcerative colitis and multiple sclerosis. The horizontal axis represents the 22 chromosomes. Colored columns represent loci showing significant local genetic correlations after multiple testing correction (*p*<0.05/1703; red for even chromosomes, blue for odd chromosomes).





**Supplementary Figure3 Sensitivity analysis of MR results**

CD: Crohn's disease; PD: Parkinson's disease. (A) Funnel plot of the MR effects of CD on PD; (B) Scatter plot of the MR effects of CD on PD; (C) Forest plot of MR leave-one-one-method effects of CD on PD; (D) Forest plot of the causal relationship between CD and PD under the IVW and MR Egger methods.

**eReferences**

1. Lloyd-Jones LR, Holloway A, McRae A, et al. The Genetic Architecture of Gene Expression in Peripheral Blood. *Am J Hum Genet*. Feb 2 2017;100(2):371. doi:10.1016/j.ajhg.2017.01.026

2. Powell JE, Henders AK, McRae AF, et al. The Brisbane Systems Genetics Study: genetical genomics meets complex trait genetics. *PLoS One*. 2012;7(4):e35430. doi:10.1371/journal.pone.0035430

3. Kim J, Ghasemzadeh N, Eapen DJ, et al. Gene expression profiles associated with acute myocardial infarction and risk of cardiovascular death. *Genome Med*. 2014;6(5):40. doi:10.1186/gm560

4. Preininger M, Arafat D, Kim J, et al. Blood-informative transcripts define nine common axes of peripheral blood gene expression. *PLoS Genet*. 2013;9(3):e1003362. doi:10.1371/journal.pgen.1003362

5. Leitsalu L, Haller T, Esko T, et al. Cohort Profile: Estonian Biobank of the Estonian Genome Center, University of Tartu. *Int J Epidemiol*. Aug 2015;44(4):1137-47. doi:10.1093/ije/dyt268

6. Idaghdour Y, Czika W, Shianna KV, et al. Geographical genomics of human leukocyte gene expression variation in southern Morocco. *Nat Genet*. Jan 2010;42(1):62-7. doi:10.1038/ng.495

7. Wu Y, Zeng J, Zhang F, et al. Integrative analysis of omics summary data reveals putative mechanisms underlying complex traits. *Nat Commun*. Mar 2 2018;9(1):918. doi:10.1038/s41467-018-03371-0

8. Kundaje A, Meuleman W, Ernst J, et al. Integrative analysis of 111 reference human epigenomes. *Nature*. Feb 19 2015;518(7539):317-30. doi:10.1038/nature14248

9. An integrated encyclopedia of DNA elements in the human genome. *Nature*. Sep 6 2012;489(7414):57-74. doi:10.1038/nature11247

10. Bulik-Sullivan B, Finucane HK, Anttila V, et al. An atlas of genetic correlations across human diseases and traits. *Nat Genet*. Nov 2015;47(11):1236-41. doi:10.1038/ng.3406

11. Shi H, Mancuso N, Spendlove S, Pasaniuc B. Local Genetic Correlation Gives Insights into the Shared Genetic Architecture of Complex Traits. *Am J Hum Genet*. Nov 2 2017;101(5):737-751. doi:10.1016/j.ajhg.2017.09.022

12. Zhu X, Feng T, Tayo BO, et al. Meta-analysis of correlated traits via summary statistics from GWASs with an application in hypertension. *Am J Hum Genet*. Jan 8 2015;96(1):21-36. doi:10.1016/j.ajhg.2014.11.011

13. Hemani G, Zheng J, Elsworth B, et al. The MR-Base platform supports systematic causal inference across the human phenome. *Elife*. May 30 2018;7doi:10.7554/eLife.34408

14. Lu Y, Quan C, Chen H, Bo X, Zhang C. 3DSNP: a database for linking human noncoding SNPs to their three-dimensional interacting genes. *Nucleic Acids Res*. Jan 4 2017;45(D1):D643-d649. doi:10.1093/nar/gkw1022

15. Giambartolomei C, Vukcevic D, Schadt EE, et al. Bayesian test for colocalisation between pairs of genetic association studies using summary statistics. *PLoS Genet*. May 2014;10(5):e1004383. doi:10.1371/journal.pgen.1004383

16. Gamazon ER, Wheeler HE, Shah KP, et al. A gene-based association method for mapping traits using reference transcriptome data. *Nat Genet*. Sep 2015;47(9):1091-8. doi:10.1038/ng.3367

17. Gusev A, Ko A, Shi H, et al. Integrative approaches for large-scale transcriptome-wide association studies. *Nat Genet*. Mar 2016;48(3):245-52. doi:10.1038/ng.3506

18. Zhu Z, Zhang F, Hu H, et al. Integration of summary data from GWAS and eQTL studies predicts complex trait gene targets. *Nat Genet*. May 2016;48(5):481-7. doi:10.1038/ng.3538
